# Corrigendum to: “The endonuclease MCPIP1 protects against liver cancer development in a sex-dependent manner by modulating β-catenin and CREB1” [JHEP Reports 8 (2026) 101755]

**DOI:** 10.1016/j.jhepr.2026.101921

**Published:** 2026-07-08

**Authors:** Oliwia Kwapisz, Paulina Marona, Judyta Górka, Rafał Myrczek, Ester Gonzalez-Sanchez, Esther Bertran, Jerzy Kotlinowski, Maciej Głuc, Ania Alay, Natalia Pydyn, Monika Kujdowicz, Emilio Ramos, Isabel Fabregat, Katarzyna Miękus

**Affiliations:** 1 Department of General Biochemistry, Faculty of Biochemistry, Biophysics and Biotechnology, Jagiellonian University, Krakow, Poland; 2 Doctoral School of Exact and Natural Sciences, Jagiellonian University, Lojasiewicza 11, 30-348, Kraków, Poland; 3 TGF-β and Cancer Group, Oncobell Program, Bellvitge Biomedical Research Institute (IDIBELL), Gran Via de l'Hospitalet, 199, 08908 Barcelona, Spain; 4 CIBEREHD, National Biomedical Research Institute on Liver and Gastrointestinal Diseases, Instituto de Salud Carlos III, Spain; 5 Unit of Bioinformatics for Precision Oncology, Institut Català d’Oncologia (ICO), L'Hospitalet de Llobregat, Barcelona, Spain(el ICO quiere que su nombre conste en catalán en los artículos); 6 Preclinical and Experimental Research in Thoracic Tumors (PReTT), Oncobell Program, Bellvitge Biomedical Research Institute (IDIBELL), L'Hospitalet de Llobregat, Barcelona, Spain; 7 Department of Pathomorphology, Faculty of Medicine, Jagiellonian University Medical College, Krakow, Grzegorzecka 16, 31-531, Poland; 8 Department of Surgery, Liver Transplant Unit, University Hospital of Bellvitge and Faculty of Medicine and Health Sciences, University of Barcelona, L'Hospitalet de Llobregat, Barcelona, Spain

It has come to our attention that the ‘Impact and implications’ section was omitted from the published version of the article. This has now been corrected online. The authors and the Editorial Office apologise for any inconvenience caused.

