# Supplementary material for: The endonuclease MCPIP1 protects against liver cancer development in a sex-dependent manner by modulating β-catenin and CREB1
Source: JHEP Rep. 2026 Jan 29;8(5):101755. doi: 10.1016/j.jhepr.2026.101755 (PMC13091202; doi:10.1016/j.jhepr.2026.101755)
Supplement: Multimedia component 1 [file mmc1.pdf]

The endonuclease MCPIP1 protects against liver cancer development in a sex-dependent manner by modulating β-catenin and the transcription factor CREB1

Oliwia Kwapisz, Paulina Marona, Judyta Gorka, Rafał Myrczek, Ester Gonzalez-Sanchez, Esther Bertran, Jerzy Kotlinowski, Maciej Głuc, Ania Alay, Natalia Pydyn, Monika Kujdowicz, Emilio Ramos, Isabel Fabregat, Katarzyna Miękus

Table of contents

Supplementary materials and methods 2

Table S1 6

Table S2 8

Fig. S1 12

Fig. S2 14

Fig. S3 16

Supplementary references 17

Editing certificates 18

Supplementary materials and methods

Immunohistochemical staining (paraffin)

To visualize liver morphology, paraffin sections were stained with Picrosirius Red and a Trichrome Stain Kit (Sigma–Aldrich). For β-catenin and c-Met staining, specific antibodies (Supplementary Table S1) and EnVision Detection System Peroxidase/DAB, Rabbit/Mouse (Dako, Agilent Technologies, Santa Clara, CA, USA) were used. All images were taken using a Leica DM6 B fluorescence microscope with 5×, 10× and 20× objectives with Leica LAS X image acquisition software.

Immunohistochemical staining (frozen)

Fresh tissues were prefixed in pure buffered formaldehyde (Chempur), washed in PBS, incubated for 12 h in 30% sucrose at 4 °C and embedded in OCT (VWR Chemicals). Then, 8-μm-thick sections were cut using a cryostat (Leica) and placed on poly-L-lysine-coated slides. Next, the sections were permeabilized (0.1% Triton X-100 in PBS) and blocked in blocking buffer (5% horse serum + 1% BSA in PBS) at room temperature for 1 h. The sections were incubated with a primary antibody in 1% BSA in PBS in a cold room. The following day, the sections were washed with PBS and incubated for 1 h at room temperature with a secondary

antibody conjugated with Alexa Fluor 488 or Alexa Fluor 546 (1:1000; Thermo Fisher Scientific) and Hoechst nuclear stain. The sections were mounted with Dako Fluorescent Mounting Medium (Agilent Technologies, cat. no. CS70330-2). Images were acquired with a Leica DM6 B fluorescence microscope with a 20× dry objective and Leica LAS X image acquisition software.

Immunofluorescence staining of cultured hepatocytes

Primary hepatocytes were seeded onto 1% collagenase-coated coverslips with a thickness of 0.17 mm, placed in the wells of a 6-well plate and fixed in 4% paraformaldehyde (Chempur) for 48 h. Coverslip cultures were permeabilized with 1% Triton X-100 in phosphate-buffered saline (PBS) and blocked with 0.2% Triton X-100 (Sigma–Aldrich) in 1% BSA in PBS. The cells were incubated with primary antibodies against β-catenin S552 (1:250), β-catenin S675 (1:100) and Creb S133 (1:250) in PBS with 1% BSA at 4°C overnight and with secondary antibodies against AlexaFluor 488 and 647 (1:1000; Thermo Fisher Scientific) for 1 h in the dark at room temperature. DNA counterstaining was performed with DAPI (Thermo Fisher Scientific). Cell imaging was conducted using a Leica Stellaris 5 confocal microscope (Leica Microsystems, Mannheim, Germany) equipped with a 63x objective (oil immersion; numerical aperture, 1.4). Alexa Fluor 647 fluorescence was excited by a 638 nm argon laser, and emission was captured using a photomultiplier that collected light within the range of 643 to 750 nm. Alexa Fluor 488 fluorescence was excited by a 488 nm argon laser, and emission was captured using a photomultiplier that collected light within the range of 502 to 712 nm. The settings for DAPI were as follows: 405 nm for excitation and 425–502 nm for emission. The confocal pinhole diameter was set to 1 Airy unit.

RNA isolation and qRT-PCR

RNA was isolated from tumor tissues using fenozol (phenol–chloroform extraction, A&A Biotechnology, Gdańsk, Poland, cat. no. 203-100). The concentration of total RNA was assessed using a NanoDrop 2000 spectrophotometer (Thermo Fisher Scientific). Reverse transcription was performed using 1 mg of total RNA, oligo(dT) 15 primer (1 µg/µl, Promega, Madison, WI, USA, cat. no. C1101), dNTPs (10 mM, Promega, cat. no. U1330) and M-MLV reverse transcriptase (Promega, cat. no. M1701). Real-time PCR was carried out using SYBRGreen Master Mix (A&A Biotechnology, cat. no. 2008-1000A) and QuantStudio 3 (Applied Biosystems, Waltham, MA, USA). Gene expression was normalized to the expression of elongation factor-2. The relative levels of transcripts were quantified by the ΔCt method. The sequences of primers (Sigma-Aldrich) and annealing temperatures are listed in

Supplementary Table S2. Additionally, for RNA-seq samples, we performed clean-up and DNAse treatment according to the manufacturer's protocol (Zymo Research).

Western blot analysis

The liver tissue was homogenized in RIPA buffer with protease and phosphatase inhibitors. Lysates were centrifugated for 20 minutes, 11,000 × *g* at 4°C.

Cellular fractionation from 1 × 10^6 hepatocytes was performed by adding hypotonic buffer (50 mM HEPES, pH 7.5, 10 mM KCl, 350 mM sucrose, 1 mM EDTA, 1 mM DTT and 0.1% Triton X-100) and incubated on ice for 10 min. After 5 min of centrifugation at 2,000 × g, the supernatant was collected as the cytoplasmic fraction. Nuclear lysate was obtained after resuspended the residual pellet in lysis buffer (10 mM HEPES, pH 7.0, 100 mM KCl, 5 mM MgCl2, 0.5% NP-40, 10 μM DTT and 1 mM PMSF). Cytoplasmic and nuclear suspension were centrifuged for 20 minutes, 14000 × rpm at 4°C to receive a high-quality nuclear and cytoplasmic fraction. SDS-PAGE was conducted with a 10% polyacrylamide gel. After wet transfer to polyvinylidene difluoride membranes (Millipore, cat. no. IPVH00010), the membranes were blocked in 3% BSA in Tris-buffered saline with 0.1% Tween 20 (Sigma-Aldrich). Next, the membranes were incubated with primary antibodies at 4 °C overnight with gentle agitation. On the following day, the membranes were washed three times for 10 min with TBS with 0.1% Tween 20 and incubated with a secondary antibody for 1 h at room temperature (RT) with gentle rocking. Chemiluminescence was detected after a 5-min incubation with Immobilon Western HRP substrate (Millipore, cat no. WBKLS0050) using a ChemiDoc system (Bio-Rad). All antibodies and dilutions are listed in Supplementary Table S1.

ELISA

Mouse DuoSet ELISA kit for IL6 (R&D Systems, cat no. DY406) were used to evaluate the levels of secreted Il6 in mice plasma, according to the manufacturer’s protocols. The absorbance was measured at 450 nm with a reference wavelength of 540 nm using a Tecan Spectre Fluor Plus microplate reader. Three independent experiments were performed in triplicate for conditioned medium experiments and without replicates for each mouse sample.

RNA-seq analysis

The transcriptome analysis was prepared by NOVOGENE (Cambridge, UK). RNA sequencing libraries were prepared using mRNA isolated from total RNA via poly-T oligo-attached magnetic beads. The mRNA was fragmented and converted to cDNA using random hexamer primers. Libraries were prepared using an unstranded workflow, including steps of end repair,

A-tailing, adapter ligation, size selection, amplification, and purification. Sequencing was performed on the NovaSeq X Plus platform (PE150). Original results have been deposited in the Sequence Read Archive (SRA) database.

Gene Set Enrichment Analysis

Gene Set Enrichment Analysis (GSEA) [1] was conducted using the GSEApy Python package [2]. The analysis was performed using a DESeq2-normalized RNA-seq dataset. The Mouse MSigDB Hallmark gene (v2023.2) set was employed for enrichment analysis [3].

Table S1

Antibody	Producent	Catalog number	Dilution
β-actin	Sigma-Aldrich	1978	1:2000
a-tubulin	Calbiochem	CP06	1:1000
Histone H3	Millipore	05-928	1:1000
NFκB	Cell Signaling Technology	4764	1:1000
phospho NFκB (Ser536)	Cell Signaling Technology	3033	1:1000
STAT3	Cell Signaling Technology	4904s	1:1000
phospho STAT3 (Tyr705)	Cell Signaling Technology	9145s	1:1000
p38	Cell Signaling Technology	9212S	1:1000
p38 (Thr180/Tyr182)	Cell Signaling Technology	9211S	1:1000
Akt	Cell Signaling Technology	9272	1:1000
Akt (Ser473)	Cell Signaling Technology	4060S	1:1000
ERK1/2 (T202/Y204)	Cell Signaling Technology	4370	1:1000
MAPK (ERK1/2)	Cell Signaling Technology	9102	1:1000
Non-phospho β-catenin (Ser45)	Cell Signaling Technology	19807	1:1000
β-catenin (Ser675)	Cell Signaling Technology	4176S	1:1000
β-catenin (Ser552)	Cell Signaling Technology	9566	1:1000

CREB1 (Ser133)	Cell Signaling Technology	9198S	1:1000
Met (Y1234/1235)	Cell Signaling Technology	3077	1:1000
Met	Santa Cruz	Sc-10	1:1000
CD45	Cell Signaling Technology	70257	1:100
a-SMA	Cell Signaling Technology	48938S	1:100
CD68	Abcam	Ab125212	1:100
Yes1	Cell Signaling Technology	3201S	1:1000
RhoA	Cell Signaling Technology	2117S	1:1000
Yap	Cell Signaling Technology	14074	1:1000
Glutamine Synthetase	Proteintech	66323-2-Ig	1:100
Anty-rabbit IgG-HRP	Santa Cruz Biotechnology	sc-2357	1:4000
Anty-mouse IgG-HRP	Santa Cruz Biotechnology	sc-516102	1:4000

Table S2

ZC3H12A	For 5'- GGAAGCAGCCGTGTCCCTATG
	Rev 5' - TCCAGGCTGCACTGCTCACTC
CTNNB1	For 5'- AAAATGGCAGTGCGTTTAG
	Rev 5' - TTTGAAGGCAGTCTGTCGTA
TGFB2	For 5'- AGATTTGCAGGTATTGATGG
	Rev 5' - ATTTCTAAAGCAATAGGCCG
SPP1	For 5'- GACCAAGGAAAACTCACTAC
	Rev 5' - CTGTTTAACTGGTATGGCAC
Zc3h12a	For 5'- CAGCCTCGACCAGATGTGCC
	Rev 5' - CAGCCGCTCCTCGATGAAGC
Ef2	For 5' - GACATCACCAAGGGTGTGCAG
	Rev 5' - TTCAGCACACTGGCATAGAGGC
Vim	For 5'- GAACCTGAGAGAAACTAACC
	Rev 5' - GATGCTGAGAAGTCTCATTG
Ctnnb1	For 5'- GATTAACTATCAGGATGACGC
	Rev 5'- TTATTAACTACCACCTGGTCC
Hgf	For 5' - CAAATGCAAGGACCTTAGAG
	Rev 5'- CTTGTTTTGGATAAGTTGCC
Fn1	For 5' - CCTATAGGATTGGAGACACG
	Rev 5'- GTTGGTAAATAGCTGTTCGG

Il1b	For 5’ - GGATGATGATGATAACCTGC
	Rev 5’- CATGGAGAATATCACTTGTTGG
Cxcl12	For 5’ - GAAAGCTTTAAACAAGAGGC
	Rev 5’- GTGAAAGTACAGCAAAACTG
Cxcr4	For 5’ - ATACTCACACTGATCGGTTC
	Rev 5’- AGGTAGCAGTGAAACCTC
Mmp2	For 5’ - GAGATCTTCTTCTTCAAGGAC
	Rev 5’- AATAGACCCAGTACTCATTCC
Zeb1	For 5’ - ATATGAGCACACAGGTAAGAG
	Rev 5’- TTCATGTGTTGAGAGTAGGAG
Twist	For 5’ - GAGACCTAGATGTCATTGTTTC
	Rev 5’- GAATTTGGTCTCTGCTCTTC
Ctgf	For 5’ - GAGGAAAACATTAAGAAGGGC
	Rev 5’- AGAAAGCTCAAACTTGACAG
Spp1	For 5’ - GGATGAATCTGACGAATCTC
	Rev 5’- GCATCAGGATACTGTTCATC
Adam17	For 5’ - AGCTTATTACAACCCAACTG
	Rev 5’- CAGCTTCCTTTGTGAGAATAG
Creb1	For 5’ - TGTGTTACGTGGGGGAGAGAA
	Rev 5’- CATGGATACCTGGGCTAATGTGG
Tgfb2	For 5’ - GAGATTTGCAGGTATTGATGG

	Rev 5'- CAACAACATTAGCAGGAGATG
Src	For 5' - AATAACACAGAGGGAGACTG
	Rev 5' - ATTCCCGTCTAGTGATCTTG
Myc	For 5' - TTTTGTCTATTTGGGGACAG
	Rev 5' - CATAGTTCCTGTTGGTGAAG
Il6	For 5' - ACTTCACAAGTCGGAGGCTT
	Rev 5' - GGTACTCCAGAAGACCAGAGG
Cd3e	For 5' - ATCTTGGTAGAGAGAGCATTC
	Rev 5' - CCCATTTTAAGTTCTCGTCAC
Cd14	For 5' - CTCTGTCCTTAAAGCGGCTTAC
	Rev 5' - GTTGCGGAGGTTCAAGATGTT
Mgl2	For 5' - AGGCACCCTAAGAGCCATTT
	Rev 5' - CCCTCTTCTCCAGTGTGCTC
Wnt4	For 5' - GTCAGGATGCTCGGACAACAT
	Rev 5' - CACGTCTTTACCTCGCAGGA
Wnt5	For 5' - CAACTGGCAGGACTTTCTCAA
	Rev 5' - CATCTCCGATGCCGGAACT
Wnt6	For 5' - GCAAGACTGGGGGTTCGAG
	Rev 5' - CCTGACAACCACACTGTAGGAG
Wnt7	For 5' - TGAACTTACACAATAACGAGGCG
	Rev 5' - GTGGTCCAGCACGTCTTAGT

Wnt11	For 5’ - ATGCGTCTACACAACAGTGAAG
	Rev 5’ - GTAGCGGGTCTTGAGGTCAG
Itpr3	For 5’ - AAGTACGGCAGCGTGATTCAG
	Rev 5’ - CACGACCACATTATCCCCATTG
Tbx3	For 5’ - GAACCTACCTGTTCCCGGAAA
	Rev 5’ - AGTGTCTCGAAAACCCTTTGC
Fzd8	For 5’ - ATGGAGTGGGTTACCTGTTG
	Rev 5’ - CACCGTGATCTCTTGGCAC
Dvl2	For 5’ - ATGGATCAGGATTTTGGGGTGG
	Rev 5’ - GTGGGGTATCTGACGACACAA
Axin	For 5’ - ATGAGTAGCGCCGTGTTAGTG
	Rev 5’ - GGGCATAGGTTTGGTGGACT
Tnfa	For 5’ - AATTCGAGTGACAAGCCTGTAGCC
	Rev 5’ - TGTCTTTGAGATCCATGCCGTTGG
Ifng	For 5’ - CAGCAAGGCGAAAAAGGATG
	Rev 5’ - CAGATACAACCCCGCAATCA
Csf2	For 5’ - TGACAGCCAGCTACTACCAG
	Rev 5’ - TCATTACGCAGGCACAAAAGC
Casp1	For 5’ - CCCACTGCTGATAGGGTGAC
	Rev 5’ - GCATAGGTACATAAGAATGAACTGGA

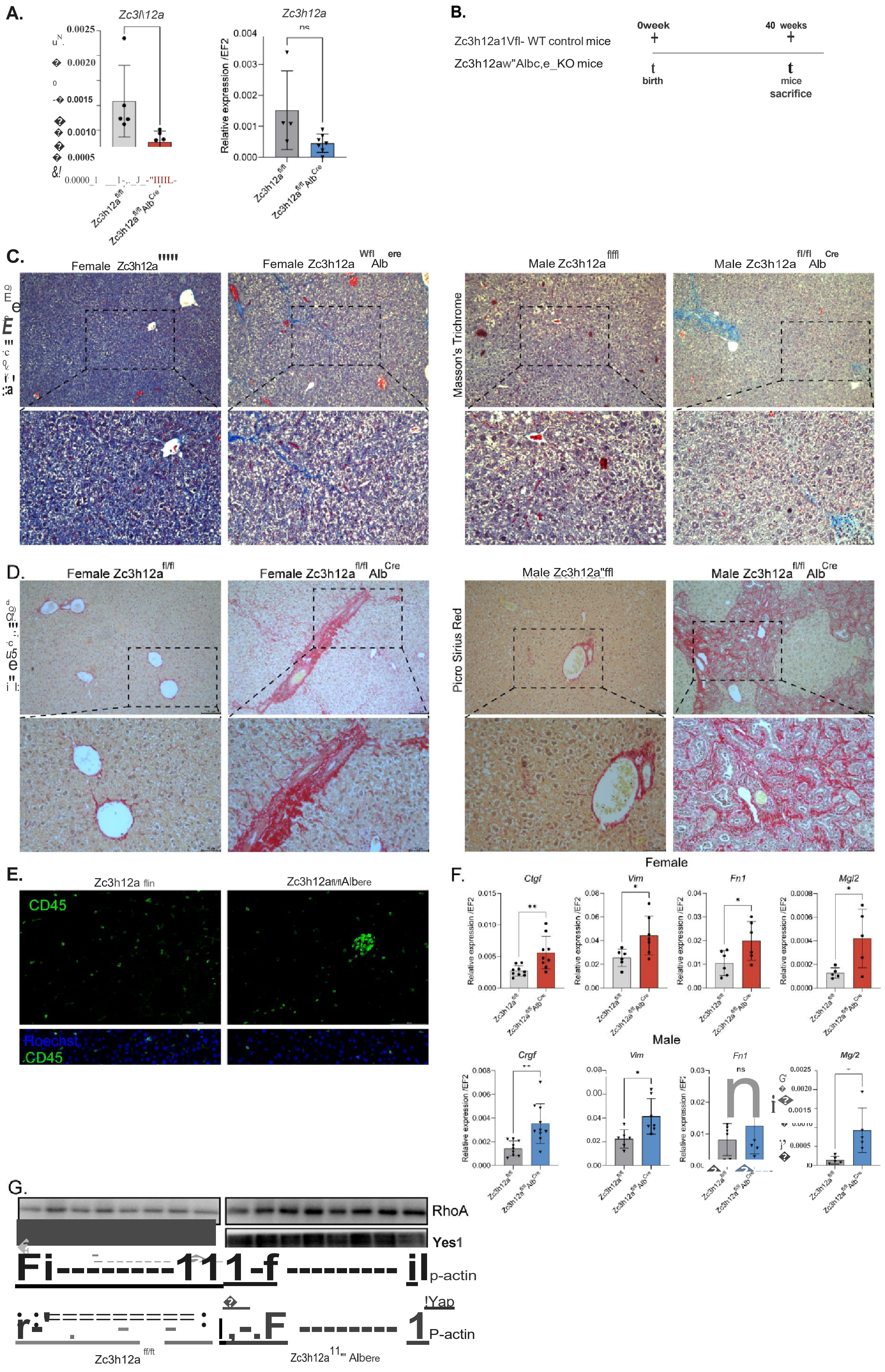
A.
B.
Oweek
40 weeks
birth
mice sacrifice
C.
Masson's Trichrome
D.
Picro Sirius Red
E.
CD45
Hoechst
F.
Female
Male
Ctgf
Vim
Fn1
Mgl2
G.
RhoA
Yes1

Fig. S1. *Zc3h12a* knock out in the liver leads to increased levels of factors involved in EMT and fibrosis. A - mRNA expression level of *Zc3h12a* in 42-weeks old male and female mice. *EF2* was used as the reference gene, N = 4-7 per group. The results are presented as the mean ± SD with dot plot. *P* values were estimated using unpaired Student's *t*-test or U Mann-Whitney $^{**}P < 0.01$.**B -** Schematic representation of the *in vivo* model without DEN administration. **C, D -** Representative images of Masson's trichrome and Picro Sirius Red staining for liver Zc3h12a$^{fl/fl}$Alb^{Cre} and Zc3h12a$^{fl/fl}$ 42 weeks old male and female mice. **E** – Representative images of CD45 immunofluorescent staining of liver 42 weeks old Zc3h12a$^{fl/fl}$Alb^{Cre} mice and Zc3h12a$^{fl/fl}$ control mice (Hoechst for nuclei; CD45 antibody labeled with fluorescent dye AlexaFluor 488). **F -** mRNA expression level of *Ctgf, Vim, Fn1,* and *Mgl2*. *EF2* was used as the reference gene. 42-weeks old males and females N = 5-10 per group. The results are presented as the mean ± SD with dot plot. *P* values were estimated using unpaired Student's *t*-test or U Mann-Whitney $^{*}P < 0.05$, $^{**}P < 0.01$. **G** – Western blot analysis of Yes1, RhoA and Yap in 42-weeks old mice with β-actin as the loading control.

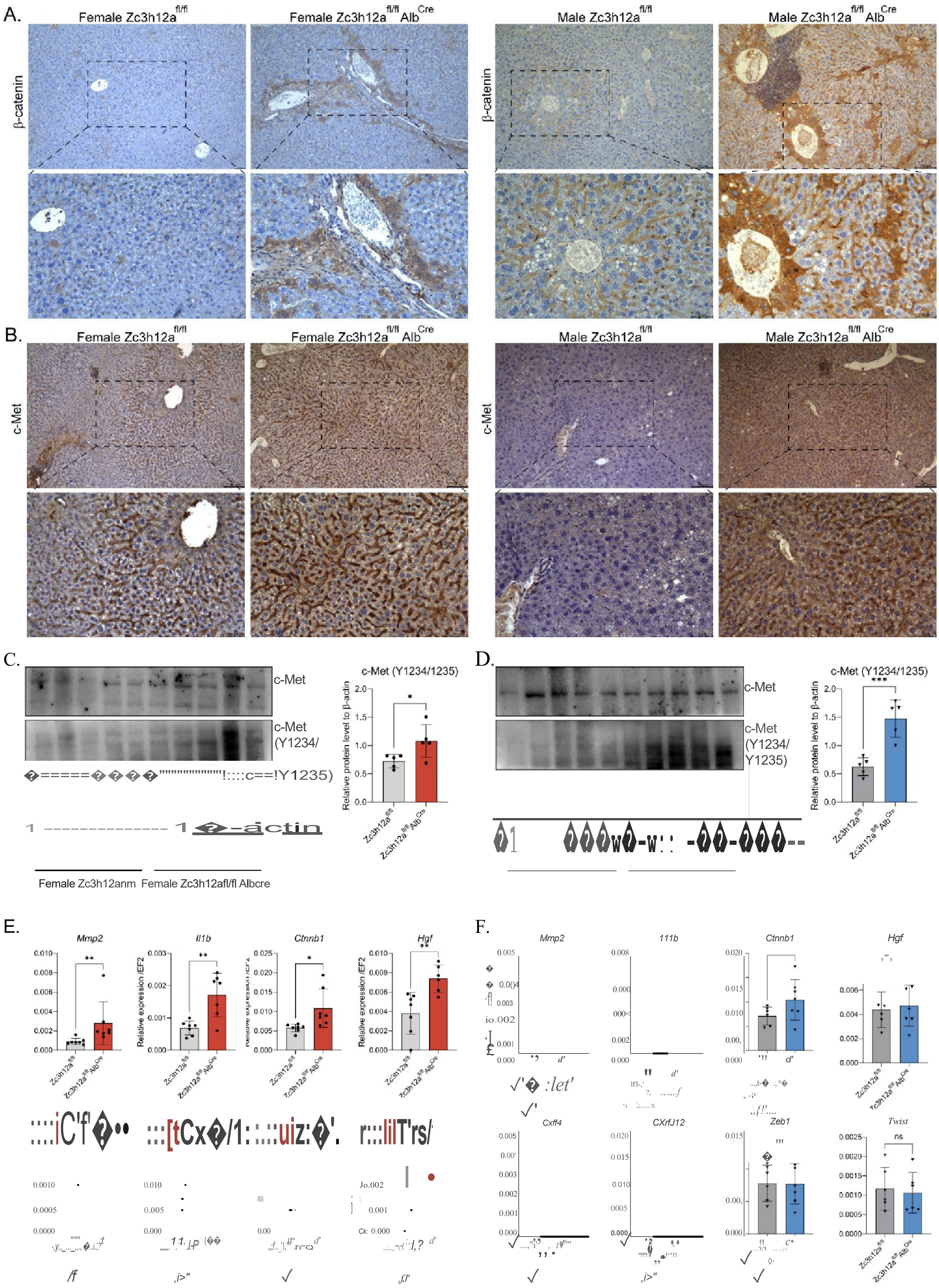
A.
Female Zc3h12a fl/fl
Female Zc3h12a fl/fl Alb Cre
Male Zc3h12a fl/fl
Male Zc3h12a fl/fl Alb Cre
β-catenin
B.
c-Met
C.
c-Met
c-Met (Y1234/
Y1235)
c-Met (Y1234/1235)
Relative protein level to β-actin
Female Zc3h12anm Female Zc3h12afl/fl Albcre
D.
c-Met
c-Met (Y1234/ Y1235)
c-Met (Y1234/1235)
Relative protein level to β-actin
E.
Mmp2
Il1b
Ctnnb1
Hgf
Relative expression /EF2
F.
Mmp2
Ctnnb1
Hgf
Cxff4
CXrfJ12
Zeb1
Twist
ns

Fig. S2. β-catenin and c-Met level is affected by Zc3h12a.

A - Representative images of β-catenin IHC staining of liver Zc3h12a$^{fl/fl}$Alb^{Cre} and Zc3h12a$^{fl/fl}$ mice (female and male). **B** – Representative images of C-Met IHC staining of liver Zc3h12a$^{fl/fl}$Alb^{Cre} and Zc3h12a$^{fl/fl}$ mice (female and male). **C, D** – Analysis of c-Met and phospho-c-Met (Y1234/Y1235) protein level in female (C, N = 5 per group) and male (D, N = 5 per group) Zc3h12a$^{fl/fl}$Alb^{Cre} and Zc3h12a$^{fl/fl}$ cmice. Graphs represent densitometric quantification with β-actin as the loading control. P values were estimated using two-tailed unpaired Student's *t*-test, *P < 0.05, ***P < 0.001. **E, F** – mRNA expression level of *Mmp2, Ctnnb1, Hgf, Zeb1, Twist*, *Il1b, Cxcl12,* and *Cxcr4*. *EF2* was used as the reference gene. Females N = 5-9 per group, males N = 5-10 per group. The results are presented as the mean ± SD with dot plot. *P* values were estimated using unpaired Student's *t*-test or U Mann-Whitney **P* < 0.05, ***P* < 0.01.

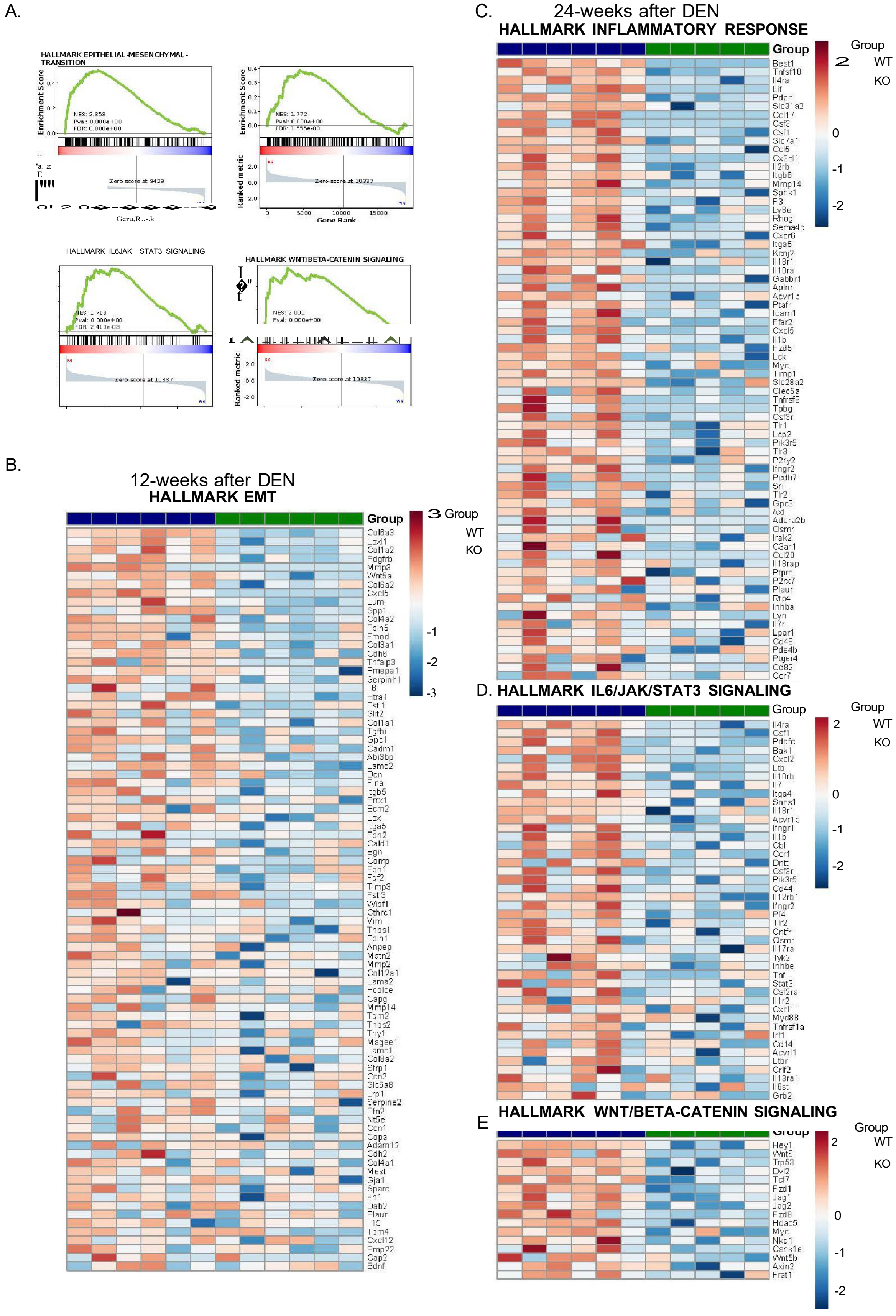
A.
HALLMARK EPITHELIAL-MESENCHYMAL-TRANSITION
HALLMARK_IL6JAK _STAT3_SIGNALING
HALLMARK WNT/BETA-CATENIN SIGNALING
B.
12-weeks after DEN
HALLMARK EMT
Group
WT
KO
C.
24-weeks after DEN
HALLMARK INFLAMMATORY RESPONSE
Group
WT
KO
D. HALLMARK IL6/JAK/STAT3 SIGNALING
Group
WT
KO
E
HALLMARK WNT/BETA-CATENIN SIGNALING
Group
WT
KO

Fig. S3. GSEA Plots and leading edge genes for Selected gene sets

A - GSEA plots for selected gene sets from an analysis performed against the mouse Hallmark gene sets from MSigDB. The plots display enrichment for upregulated pathways in knockout mice. The y-axis represents the enrichment score (ES), while the x-axis indicates the ranked position of all genes included in the analysis. The peak of each curve corresponds to the maximum ES, calculated by walking down the ranked gene list. Genes contributing most to the enrichment signal before this peak are defined as the 'leading edge' subset. **B, C, D, E** - Corresponding heatmaps show the expression of leading-edge genes, based on normalized RNA-seq read counts.

Supplementary references

1. Subramanian A, Tamayo P, Mootha VK, et al. Gene set enrichment analysis: A knowledge-based approach for interpreting genome-wide expression profiles. *Proceedings of the National Academy of Sciences*. 2005;102(43):15545-15550. doi:10.1073/pnas.0506580102
2. Fang Z, Liu X, Peltz G. GSEApy: a comprehensive package for performing gene set enrichment analysis in Python. *Bioinformatics*. 2023;39(1). doi:10.1093/bioinformatics/btac757
3. Liberzon A, Birger C, Thorvaldsdóttir H, Ghandi M, Mesirov JP, Tamayo P. The Molecular Signatures Database Hallmark Gene Set Collection. *Cell Syst*. 2015;1(6):417-425. doi:10.1016/j.cels.2015.12.004

SPRINGER NATURE

Author Services

Editing Certificate

This document certifies that the manuscript

Endonuclease MCPIP1 protects against liver cancer development in a gender-dependent manner by modulating β-Catenin and the transcription factor CREB1

prepared by the authors

Oliwia Kwapisz, Paulina Marona, Judyta Gorka, Rafał Myrczek, Ester Gonzalez-Sanchez, Esther Bertran, Jerzy Kotlinowski, Maciej Głuc, Ania Alay, Natalia Pydyn, Monika Kujdowicz, Emilio Ramos, Isabel Fabregat, Katarzyna Miekus

was edited for proper English language, grammar, punctuation, spelling, and overall style by one or more of the highly qualified English speaking editors at SNAS.

This certificate was issued on **April 29, 2025** and may be verified on the SNAS website using the verification code **3401-F1BA-84B9-4DA0-10DP**.

Neither the research content nor the authors' intentions were altered in any way during the editing process. Documents receiving this certification should be English-ready for publication; however, the author has the ability to accept or reject our suggestions and changes. To verify the final SNAS edited version, please visit our verification page at secure.authorservices.springernature.com/certificate/verify.
If you have any questions or concerns about this edited document, please contact SNAS at support@as.springernature.com.

SNAS provides a range of editing, translation, and manuscript services for researchers and publishers around the world.
For more information about our company, services, and partner discounts, please visit authorservices.springernature.com.

SPRINGER NATURE

Author Services

Editing Certificate

This document certifies that the manuscript

The endonuclease MCPIP1 protects against liver cancer development in a sex-dependent manner by modulating β-catenin and the transcription factor CREB1

prepared by the authors

Oliwia Kwapisz, Paulina Marona, Judyta Gorka, Rafał Myrczek, Ester Gonzalez-Sanchez, Esther Bertran, Jerzy Kotlinowski, Maciej Głuc, Ania Alay, Natalia Pydyn, Monika Kujdowicz, Emilio Ramos, Isabel Fabregat, Katarzyna Miekus

was edited for proper English language, grammar, punctuation, spelling, and overall style by one or more of the highly qualified English speaking editors at SNAS.

This certificate was issued on **December 17, 2025** and may be verified on the SNAS website using the verification code **CFC3-F3B2-B2C2-B9B3-C025**.

Neither the research content nor the authors' intentions were altered in any way during the editing process. Documents receiving this certification should be English-ready for publication; however, the author has the ability to accept or reject our suggestions and changes. To verify the final SNAS edited version, please visit our verification page at secure.authorservices.springernature.com/certificate/verify.
If you have any questions or concerns about this edited document, please contact SNAS at support@as.springernature.com.

SNAS provides a range of editing, translation, and manuscript services for researchers and publishers around the world.
For more information about our company, services, and partner discounts, please visit authorservices.springernature.com.
