# Supplementary material for: The endonuclease MCPIP1 protects against liver cancer development in a sex-dependent manner by modulating β-catenin and CREB1
Source: JHEP Rep. 2026 Jan 29;8(5):101755. doi: 10.1016/j.jhepr.2026.101755 (PMC13091202; doi:10.1016/j.jhepr.2026.101755)
Supplement: Multimedia component 2 [file mmc2.docx]

**JHEP Reports**

**CTAT methods**

Tables for a “Complete, Transparent, Accurate and Timely account” (CTAT) are now mandatory for all revised submissions. The aim is to enhance the reproducibility of methods.

- Only include the parts relevant to your study
- Refer to the CTAT in the main text as ‘Supplementary CTAT Table’
- Do not add subheadings
- Add as many rows as needed to include all information
- Only include one item per row

**If the CTAT form is not relevant to your study, please outline the reasons why:**

|  |
| --- |

- 1. **Antibodies**

| **Name** | **Citation** | **Supplier** | **Cat no.** | **Clone no.** |
| --- | --- | --- | --- | --- |
| β-actin | Manufacturer’s data sheet | Sigma-Aldrich | 1978 | Ms mAb AC-15 |
| α-tubulin | Manufacturer’s data sheet | Calbiochem | CP06 | N/A |
| Histone H3 | Manufacturer’s data sheet | Millipore | 05-928 | A3S |
| NFκB | Manufacturer’s data sheet | Cell Signaling Technology | 4764 | C22B4 |
| phospho NFκB  (Ser536) | Manufacturer’s data sheet | Cell Signaling Technology | 3033 | 93H1 |
| STAT3 | Manufacturer’s data sheet | Cell Signaling Technology | 4904s | 79D7 |
| phospho STAT3 (Tyr705) | Manufacturer’s data sheet | Cell Signaling Technology | 9145s | D3A7 |
| p38 | Manufacturer’s data sheet | Cell Signaling Technology | 9212S | N/A |
| p38 (Thr180/Tyr182) | Manufacturer’s data sheet | Cell Signaling Technology | 9211S | N/A |
| Akt | Manufacturer’s data sheet | Cell Signaling Technology | 9272 | N/A |
| Akt (Ser473) | Manufacturer’s data sheet | Cell Signaling Technology | 4060S | Rb mAb D9E |
| ERK1/2 (T202/Y204) | Manufacturer’s data sheet | Cell Signaling Technology | 9101S | N/A |
| MAPK (ERK1/2) | Manufacturer’s data sheet | Cell Signaling Technology | 9102 | N/A |
| Non-phospho β-catenin (Ser45) | Manufacturer’s data sheet | Cell Signaling Technology | 19807 | Rb mAb D2U8Y |
| β-catenin (Ser675) | Manufacturer’s data sheet | Cell Signaling Technology | 4176S | D2F1 |
| β-catenin (Ser552) | Manufacturer’s data sheet | Cell Signaling Technology | 9566 | N/A |
| CREB1 (Ser133) | Manufacturer’s data sheet | Cell Signaling Technology | 9198S | 87G3 |
| Met (Y1234/1235) | Manufacturer’s data sheet | Cell Signaling Technology | 3077 | Rb mAb D26 |
| Met | Manufacturer’s data sheet | Santa Cruz | Sc-10 | C-12 |
| CD45 | Manufacturer’s data sheet | Cell Signaling Technology | 70257 | D3F8Q |
| α-SMA | Manufacturer’s data sheet | Cell Signaling Technology | 19245S | D4K9N |
| CD68 | Manufacturer’s data sheet | Abcam | Ab125212 | 1060297-10 |
| Yes1 | Manufacturer’s data sheet | Cell Signaling Technology | 3201S | N/A |
| RhoA | Manufacturer’s data sheet | Cell Signaling Technology | 2117S | Rb mAb 67B9 |
| Yap | Manufacturer’s data sheet | Cell Signaling Technology | 14074 | D8H1X |
| Glutamine Synthetase | Manufacturer’s data sheet | Proteintech | 66323-2-Ig | N/A |
| Anty-rabbit IgG-HRP | Manufacturer’s data sheet | Santa Cruz Biotechnology | sc-2357 | N/A |
| Anty-mouse IgG-HRP | Manufacturer’s data sheet | Santa Cruz Biotechnology | sc-516102 | N/A |

- 1. **Cell lines**

| **Name** | **Citation** | **Supplier** | **Cat no.** | **Passage no.** | **Authentication test method** |
| --- | --- | --- | --- | --- | --- |
| Not applicable |  |  |  |  |  |

- 1. **Organisms**

| **Name** | **Citation** | **Supplier** | **Strain** | **Sex** | **Age** | **Overall n number** |
| --- | --- | --- | --- | --- | --- | --- |
| Mouse | Kotlinowski J, et al. Deletion of Mcpip1 in  Mcpip1fl/flAl  bCre mice  recapitulates  the phenotype  of human primary biliary  cholangitis.  Biochim Biophys Acta Mol Basis Dis.  2021;1867(5). | Jagiellonian University:  B6(Cg)-  Zc3h12atm1c(EUCOMM)Hm  gu/FumJ  crossed with liver-specific  Cre-expressing transgenic  mice (AlbCre tg/+), (Jackson Laboratory) | Zc3h12a^fl/fl^ | Female | 3 months | 9 |
| Mouse | as above | as above | Zc3h12a^fl/fl^Alb^Cre^ | Female | 3 months | 7 |
| Mouse | as above | as above | Zc3h12a^fl/fl^ | Male | 3 months | 7 |
| Mouse | as above | as above | Zc3h12a^fl/fl^Alb^Cre^ | Male | 3 months | 9 |
| Mouse | as above | as above | Zc3h12a^fl/fl^ | Female | 6 months | 6 |
| Mouse | as above | as above | Zc3h12a^fl/fl^Alb^Cre^ | Female | 6 months | 8 |
| Mouse | as above | as above | Zc3h12a^fl/fl^ | Male | 6 months | 3 |
| Mouse | as above | as above | Zc3h12a^fl/fl^Alb^Cre^ | Male | 6 months | 4 |
| Mouse | as above | as above | Zc3h12a^fl/fl^ | Female | 10 months | 26 |
| Mouse | as above | as above | Zc3h12a^fl/fl^Alb^Cre^ | Female | 10 months | 28 |
| Mouse | as above | as above | Zc3h12a^fl/fl^ | Male | 10 months | 31 |
| Mouse | as above | as above | Zc3h12a^fl/fl^Alb^Cre^ | Male | 10 months | 31 |

- 1. **Sequence based reagents**

| **Name** | **Sequence** | **Supplier** |
| --- | --- | --- |
| *ZC3H12A* | For 5’- GGAAGCAGCCGTGTCCCTATG | Sigma Aldrich |
|  | Rev 5’ - TCCAGGCTGCACTGCTCACTC |  |
| *CTNNB1* | For 5’- AAAATGGCAGTGCGTTTAG | Sigma Aldrich |
|  | Rev 5’ - TTTGAAGGCAGTCTGTCGTA |  |
| *TGFB2* | For 5’- AGATTTGCAGGTATTGATGG | Sigma Aldrich |
|  | Rev 5’ - ATTTCTAAAGCAATAGGCCG |  |
| *SPP1* | For 5’- GACCAAGGAAAACTCACTAC | Sigma Aldrich |
|  | Rev 5’ - CTGTTTAACTGGTATGGCAC |  |
| *Zc3h12a* | For 5’- CAGCCTCGACCAGATGTGCC | Sigma Aldrich |
|  | Rev 5’ - CAGCCGCTCCTCGATGAAGC |  |
| *Ef2* | For 5’ - GACATCACCAAGGGTGTGCAG | Sigma Aldrich |
|  | Rev 5’ - TTCAGCACACTGGCATAGAGGC |  |
| *Vim* | For 5’- GAACCTGAGAGAAACTAACC | Sigma Aldrich |
|  | Rev 5’ - GATGCTGAGAAGTCTCATTG |  |
| *Ctnnb1* | For 5’- GATTAACTATCAGGATGACGC | Sigma Aldrich |
|  | Rev 5’- TTATTAACTACCACCTGGTCC |  |
| *Hgf* | For 5’ - CAAATGCAAGGACCTTAGAG | Sigma Aldrich |
|  | Rev 5’- CTTGTTTTGGATAAGTTGCC |  |
| *Fn1* | For 5’ - CCTATAGGATTGGAGACACG | Sigma Aldrich |
|  | Rev 5’- GTTGGTAAATAGCTGTTCGG |  |
| *Il1b* | For 5’ - GGATGATGATGATAACCTGC | Sigma Aldrich |
|  | Rev 5’- CATGGAGAATATCACTTGTTGG |  |
| *Cxcl12* | For 5’ - GAAAGCTTTAAACAAGAGGC | Sigma Aldrich |
|  | Rev 5’- GTGAAAGTACAGCAAAACTG |  |
| *Cxcr4* | For 5’ - ATACTCACACTGATCGGTTC | Sigma Aldrich |
|  | Rev 5’- AGGTAGCAGTGAAACCTC |  |
| *Mmp2* | For 5’ - GAGATCTTCTTCTTCAAGGAC | Sigma Aldrich |
|  | Rev 5’- AATAGACCCAGTACTCATTCC |  |
| *Zeb1* | For 5’ - ATATGAGCACACAGGTAAGAG | Sigma Aldrich |
|  | Rev 5’- TTCATGTGTTGAGAGTAGGAG |  |
| *Twist* | For 5’ - GAGACCTAGATGTCATTGTTTC | Sigma Aldrich |
|  | Rev 5’- GAATTTGGTCTCTGCTCTTC |  |
| *Ctgf* | For 5’ - GAGGAAAACATTAAGAAGGGC | Sigma Aldrich |
|  | Rev 5’- AGAAAGCTCAAACTTGACAG |  |
| *Spp1* | For 5’ - GGATGAATCTGACGAATCTC | Sigma Aldrich |
|  | Rev 5’- GCATCAGGATACTGTTCATC |  |
| *Adam17* | For 5’ - AGCTTATTACAACCCAACTG | Sigma Aldrich |
|  | Rev 5’- CAGCTTCCTTTGTGAGAATAG |  |
| *Creb1* | For 5’ - TGTGTTACGTGGGGGAGAGAA | Sigma Aldrich |
|  | Rev 5’- CATGGATACCTGGGCTAATGTGG |  |
| *Tgfb2* | For 5’ - GAGATTTGCAGGTATTGATGG | Sigma Aldrich |
|  | Rev 5’- CAACAACATTAGCAGGAGATG |  |
| *Src* | For 5’ - AATAACACAGAGGGAGACTG | Sigma Aldrich |
|  | Rev 5’ - ATTCCCGTCTAGTGATCTTG |  |
| *Myc* | For 5’ - TTTTGTCTATTTGGGGACAG | Sigma Aldrich |
|  | Rev 5’ - CATAGTTCCTGTTGGTGAAG |  |
| *Il6* | For 5’ - ACTTCACAAGTCGGAGGCTT | Sigma Aldrich |
|  | Rev 5’ - GGTACTCCAGAAGACCAGAGG |  |
| *Cd3e* | For 5’ - ATCTTGGTAGAGAGAGCATTC | Sigma Aldrich |
|  | Rev 5’ - CCCATTTTAAGTTCTCGTCAC |  |
| *Cd14* | For 5’ - CTCTGTCCTTAAAGCGGCTTAC | Sigma Aldrich |
|  | Rev 5’ - GTTGCGGAGGTTCAAGATGTT |  |
| *Mgl2* | For 5’ - AGGCACCCTAAGAGCCATTT | Sigma Aldrich |
|  | Rev 5’ - CCCTCTTCTCCAGTGTGCTC |  |
| *Wnt4* | For 5’ - GTCAGGATGCTCGGACAACAT | Sigma Aldrich |
|  | Rev 5’ - CACGTCTTTACCTCGCAGGA |  |
| *Wnt5* | For 5’ - CAACTGGCAGGACTTTCTCAA | Sigma Aldrich |
|  | Rev 5’ - CATCTCCGATGCCGGAACT |  |
| *Wnt6* | For 5’ - GCAAGACTGGGGGTTCGAG | Sigma Aldrich |
|  | Rev 5’ - CCTGACAACCACACTGTAGGAG |  |
| *Wnt7* | For 5’ - TGAACTTACACAATAACGAGGCG | Sigma Aldrich |
|  | Rev 5’ - GTGGTCCAGCACGTCTTAGT |  |
| *Wnt11* | For 5’ - ATGCGTCTACACAACAGTGAAG | Sigma Aldrich |
|  | Rev 5’ - GTAGCGGGTCTTGAGGTCAG |  |
| *Itpr3* | For 5’ - AAGTACGGCAGCGTGATTCAG | Sigma Aldrich |
|  | Rev 5’ - CACGACCACATTATCCCCATTG |  |
| *Tbx3* | For 5’ - GAACCTACCTGTTCCCGGAAA | Sigma Aldrich |
|  | Rev 5’ - AGTGTCTCGAAAACCCTTTGC |  |
| *Fzd8* | For 5’ - ATGGAGTGGGTTACCTGTTG | Sigma Aldrich |
|  | Rev 5’ - CACCGTGATCTCTTGGCAC |  |
| *Dvl2* | For 5’ - ATGGATCAGGATTTTGGGGTGG | Sigma Aldrich |
|  | Rev 5’ - GTGGGGTATCTGACGACACAA |  |
| *Axin* | For 5’ - ATGAGTAGCGCCGTGTTAGTG | Sigma Aldrich |
|  | Rev 5’ - GGGCATAGGTTTGGTGGACT |  |
| *Tnfa* | For 5’ - AATTCGAGTGACAAGCCTGTAGCC | Sigma Aldrich |
|  | Rev 5’ - TGTCTTTGAGATCCATGCCGTTGG |  |
| *Ifng* | For 5’ - CAGCAAGGCGAAAAAGGATG | Sigma Aldrich |
|  | Rev 5’ - CAGATACAACCCCGCAATCA |  |
| *Csf2* | For 5’ - TGACAGCCAGCTACTACCAG | Sigma Aldrich |
|  | Rev 5’ - TCATTACGCAGGCACAAAAGC |  |
| *Casp1* | For 5’ - CCCACTGCTGATAGGGTGAC | Sigma Aldrich |
|  | Rev 5’ - GCATAGGTACATAAGAATGAACTGGA |  |
| miR-200a-3p | ​ Mature miRNA sequence ​UAACACUGUCUGGUAACGAUGU | Qiagen |
| miR-103-3p | Mature miRNA sequence​ AGCAGCAUUGUACAGGGCUAUGA | Qiagen |

- 1. **Biological samples**

| **Description** | **Source** | **Identifier** |
| --- | --- | --- |
| Samples from tumor (T) of histological grade 1 or 2 and adjacent nontumor (NT) liver tissues | Bellvitge University Hospital | Comité Ético de Investigación Clínica‐CEIC, University Hospital of Bellvitge; approval number PR202/22 |

- 1. **Deposited data**

| **Name of repository** | **Identifier** | **Link** |
| --- | --- | --- |
| Sequence Read Archive | PRJNA1198596 | https://www.ncbi.nlm.nih.gov/bioproject/1198596 |

- 1. **Software**

| **Software name** | **Manufacturer** | **Version** |
| --- | --- | --- |
| GraphPad Prism | GraphPad Software, Inc. | 10 |
| Image Lab | Bio-Rad | 4.1 |
| QuantStudio Design and Analysis | Applied Biosystem | V1.5.x |
| hisat2 | DOI: 10.1101/gr.275193.120 | 2.0.5 |
| featureCounts | DOI: 10.1093/bioinformatics/btt656 | 1.5.0-p3 |
| deseq2 | DOI https://doi.org/10.1186/s13059-014-0550-8 | 1.42.0 |
| gseapy | https://doi.org/10.1093/bioinformatics/btac757 | 1.1.2 |
| pheatmap | https://github.com/raivokolde/pheatmap. | 1.0.12 |

- 1. **Other (*e.g*. drugs, proteins, vectors etc.)**

| Diethylnitrosamine (DEN) | Sigma-Aldrich |  |
| --- | --- | --- |

- 1. **Please provide the details of the corresponding methods author for the manuscript:**

| Katarzyna Miekus, PhD  E-mail: katarzyna.miekus@uj.edu.pl  Department of General Biochemistry, Faculty of Biochemistry, Biophysics and Biotechnology, Jagiellonian University, Gronostajowa Street 7, Krakow, Poland |
| --- |

**2.0 Please confirm for randomised controlled trials all versions of the clinical protocol are included in the submission. These will be published online as supplementary information.**

| Not applicable (this study did not include a randomized controlled trial) |
| --- |
