# Supplementary material for: The endonuclease MCPIP1 protects against liver cancer development in a sex-dependent manner by modulating β-catenin and CREB1
Source: JHEP Rep. 2026 Jan 29;8(5):101755. doi: 10.1016/j.jhepr.2026.101755 (PMC13091202; doi:10.1016/j.jhepr.2026.101755)
Supplement: Multimedia component 4 [file mmc4.pdf]

Research article

JHEP|Reports

# The endonuclease MCPIP1 protects against liver cancer development in a sex-dependent manner by modulating β-catenin and CREB1


## Authors

**Oliwia Kwapisz, Paulina Marona,** Judyta Gorka, …, Emilio Ramos, Isabel Fabregat, Katarzyna Miekus

## Correspondence

katarzyna.miekus@uj.edu.pl (K. Miekus).


## Graphical abstract

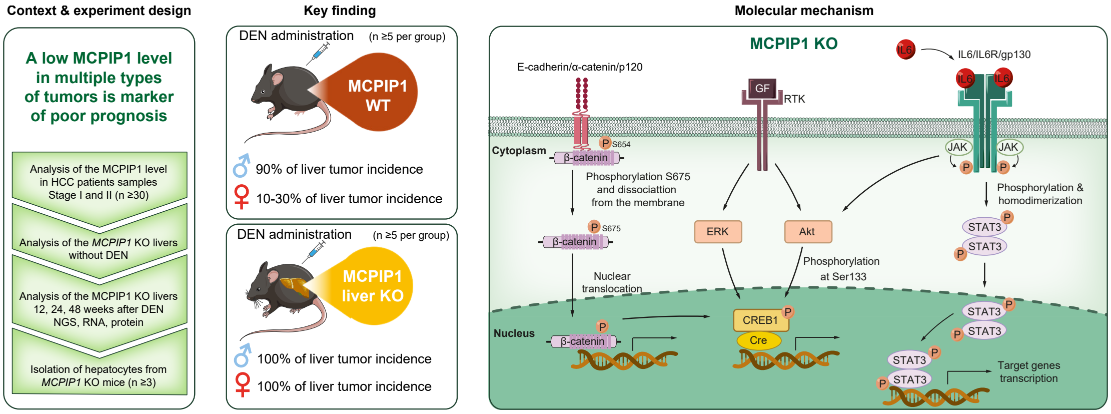


## Highlights:

- MCPIP1 deficiency triggers liver fibrosis, inflammation, and immune cell infiltration.
- Loss of MCPIP1 promotes hepatocellular carcinoma development in female mice.
- MCPIP1 regulates Wnt/β-catenin, STAT3, and NF-κB signaling in hepatocytes.
- MCPIP1 knockout activates β-catenin and CREB1, promoting malignant transformation.

https://doi.org/10.1016/j.jhepr.2026.101755

© 2026 The Author(s). Published by Elsevier B.V. on behalf of European Association for the Study of the Liver (EASL). This is an open access article under the CC BY license (http://creativecommons.org/licenses/by/4.0/). JHEP Reports, 2026, 8, 1–15

# The endonuclease MCPIP1 protects against liver cancer development in a sex-dependent manner by modulating β-catenin and CREB1

**Oliwia Kwapisz**[1,2,†], **Paulina Marona**[1,†], Judyta Gorka[1], Rafał Myrczek[1,2], Ester Gonzalez-Sanchez[3,4,&], Esther Bertran[3,4], Jerzy Kotlinowski[1], Maciej Głuc[1], Ania Alay[5,6], Natalia Pydyn[1], Monika Kujdowicz[7], Emilio Ramos[3,4,8], Isabel Fabregat[3,4], Katarzyna Miekus[1,*]

JHEP Reports **2026**. vol. 8 | 1–15

Check for updates

**Background & Aims:** Monocyte chemoattractant protein-induced protein 1 (MCPIP1), encoded by *ZC3H12A*, is a negative regulator of inflammation and tumorigenesis. While its role has been implicated in various cancers, the function of MCPIP1 in hepatocellular carcinoma (HCC) remains poorly understood. This study explored the contribution of hepatocyte-specific MCPIP1 loss to HCC pathogenesis, highlighting its role in overcoming the inherent tumor resistance observed in female mice.

**Methods:** Liver tissues (n ≥5 per group) and primary hepatocytes (n ≥3 per group) were evaluated using western blotting, immunohistochemistry, immunofluorescence, RNA sequencing and pathway-enrichment analysis. The expression levels of MCPIP1 in HCC were measured by quantitative reverse-transcription PCR. The results are presented as mean ± SD, Student's *t* or Mann−Whitney *U* tests were used for statistical analysis of two groups. For more than two groups, ordinary two-way ANOVA was used.

**Results:** The hepatocyte-specific loss of MCPIP1 markedly promoted fibrosis and tumorigenesis, particularly in female mice, disrupting the normal sex-related protection observed in the diethylnitrosamine model. As determined by next-generation sequencing and bioinformatics analysis, oncogenic and fibrotic programs, including the EMT, Wnt/β-catenin, and JAK/STAT3 pathways, were activated in MCPIP1 knockout livers. These molecular events activated β-catenin, c-Met, and IL-6/STAT3/NF-κB signaling, and they enhanced fibrotic remodeling. In MCPIP1-deficient hepatocytes, active β-catenin and CREB1 accumulated in the nucleus, and the expression of protumorigenic targets, such as *Spp1*, *Tgfb2*, and *Adam17* increased. Moreover, MCPIP1 expression was significantly downregulated in human HCC tissues and correlated with tumor progression.

**Conclusions:** MCPIP1 plays a protective role against inflammation-driven hepatocarcinogenesis, particularly in females, by restraining fibrotic remodeling and oncogenic signaling. The downregulation of MCPIP1 expression promotes a tumor-promoting microenvironment through the coordinated activation of the β-catenin, STAT3, and CREB1 pathways.

© 2026 The Author(s). Published by Elsevier B.V. on behalf of European Association for the Study of the Liver (EASL). This is an open access article under the CC BY license (http://creativecommons.org/licenses/by/4.0/).

## Introduction

Hepatocellular carcinoma (HCC) is among the most prevalent and clinically challenging malignancies worldwide, with the annual number of cases anticipated to surpass one million by 2025.[1] In approximately 90% of patients, HCC is diagnosed at an advanced stage, making curative surgery difficult and contributing to a consistently poor prognosis, with a 5-year survival rate of only 18%.[2,3] Therapeutic options are limited, with the sorafenib multikinase inhibitor showing a survival benefit of only 3 months.[3] Therefore, there is a critical need for earlier diagnosis and more effective treatment options for HCC.

Compared with women, men are at a significantly greater risk of developing HCC, with an approximately three to five times increased risk. This sex disparity is also evident in rodent models of HCC. Estrogen administration in male mice has been shown to suppress the development of chemically induced HCC.[4] However, the mechanisms underlying this sex difference and the anticancer effects of estrogen remain poorly understood. The administration of diethylnitrosamine (DEN) to mice leads to increased IL-6 concentrations in males, this response is suppressed in females by estrogens.[4]

Interleukin 6 (IL-6) is among the main inducers of inflammation and is a catalyst for DNA damage, mutagenesis, hepatic cell death, and compensatory proliferation.[4–6] This inflammatory environment is closely associated with the hyperactivation of key signaling pathways, including the NF-κB, MAPK, STAT3, and AKT pathways, which are essential for HCC development.[6–9] Moreover, tumor promotion depends on interactions between initiated cells and their microenvironment, which exerts

* Corresponding author. Address: Department of General Biochemistry, Faculty of Biochemistry, Biophysics and Biotechnology, Jagiellonian University, Gronostajowa Street 7, Krakow, Poland.
*E-mail address:* katarzyna.miekus@uj.edu.pl (K. Miekus).
† Contributed equally.
& Current address: Department of Physiology and Pharmacology, University of Salamanca, 37007, Salamanca, Spain. HepatoBiliary Tumours Lab, Centro de Investigación del Cáncer and Instituto de Biología Molecular y Celular del Cáncer, CSIC-Universidad de Salamanca, Salamanca 37007, Spain.
https://doi.org/10.1016/j.jhepr.2026.101755

constant evolutionary pressure on early neoplastic cells through the production of proinflammatory cytokines, chemokines, and reactive oxygen species.[7] Understanding the molecular mechanisms underlying the malignant conversion of premalignant lesions during HCC development is critical for delaying or preventing HCC development.

Monocyte chemoattractant protein-induced protein 1 (MCPIP1 or *ZC3H12A*, also known as Regnase-1) has emerged as a key regulator of the inflammatory response and negatively modulates cellular inflammation. The primary function of MCPIP1 involves binding to stem–loop structures in the 3′ untranslated regions of major proinflammatory cytokines (IL-6, IL-1β, and IL-12b).[10] Additionally, MCPIP1 negatively regulates JNK and NF-κB activity,[11] indicating its potential to regulate cancer-associated features, including proliferation, angiogenesis, growth arrest, and the modulation of a proinflammatory microenvironment.

Moreover, we have previously reported that MCPIP1 expression is reduced in patients with MASLD (metabolic dysfunction-associated steatotic liver disease) and that MCPIP1 inhibits hepatic stellate cell activation.[12,13] However, the mechanism through which MCPIP1 regulates HCC development remains unexplored.

For the first time, the present study demonstrated that MCPIP1 protects against HCC development in females, acting as a necessary second hit for HCC occurrence. A time-dependent transcriptomic analysis of tumor initiation and development revealed that the absence of MCPIP1 first changes the set of genes important for K-Ras signaling, angiogenesis and epithelial-to-mesenchymal transition (EMT), as well as the genes important for β-catenin signaling. Moreover, the absence of MCPIP1 in hepatocytes triggers the expression of *Ctgf*, *Mmp2*, *Hgf*, *Tgfb2*, *Spp1* and *Il6*, which are integral to tumor development and immune cell activation, thus promoting significant fibrotic alterations and enhanced tissue remodeling in the liver. Moreover, a lack of MCPIP1 leads to β-catenin and cAMP-responsive element-binding protein 1 (CREB1) activation, which is crucial for HCC development. The present study revealed that liver-specific MCPIP1 knockout has notable molecular consequences, indicating that MCPIP1 plays a protective role during hepatocarcinogenesis.

## Materials and methods

### Patient samples

Samples from tumor and adjacent non-tumor liver tissues were obtained from patients during surgical procedures at Bellvitge University Hospital (HUB). The samples were of histological grade 1 or 2. Human tissues were collected after receiving the required written informed consent from each patient and with the approval of the Institutional Review Board (Comité Ético de Investigación Clínica-CEIC, University Hospital of Bellvitge; approval number PR202/22). Patients provided written consent, and the study protocol conformed to the ethical guidelines of the 1975 Declaration of Helsinki.

### Animal studies

Animal experiments were conducted in accordance with the Institutional Animal Care and II Local Ethics Committee of the Institute of Pharmacology, Polish Academy of Sciences (approval numbers 254/2018, 112/2023 and 935A/2024). The mice were handled in accordance with the regulations of national and local animal welfare under specific pathogen-free conditions, and they were provided water and food *ad libitum*. Two-week-old mice were intraperitoneally administered diethylnitrosamine (DEN; Sigma–Aldrich, St. Louis, MO, USA) dissolved in NaCl at a concentration of 25 mg/kg body weight. Tissues were collected 40 weeks after birth and 12, 24, or 40 weeks after DEN administration. Liver lobes were divided for RNA and protein isolation (lobus hepatis sinister medialis and lobus hepatis caudatus for reserve) and histology (lobus hepatis sinister lateralis and dexter lateralis).

### Primary hepatocyte isolation and culture

Primary hepatocytes were isolated from *Zc3h12a*$^{fl/fl}$ and *Zc3h12a*$^{fl/fl}$*Alb*$^{Cre}$ mice via collagenase perfusion as described previously.[14] Briefly, the animals were anesthetized with ketamine (100 mg/kg) and xylazine (10 mg/kg) administered intraperitoneally. Next, the livers were perfused via the inferior vena cava with 20 ml of Krebs-Ringer buffer supplemented with 0.1 mM EGTA, followed by 25 ml of digestion solution (Krebs-Ringer containing 4.76 mM $CaCl_2$ and 200 U/ml collagenase IV (Gibco)). After the liver was excised, it was disrupted in a Petri dish containing 10 ml of complete medium and filtered through a 100 µm cell strainer. The cells were subsequently centrifuged (*50 × g*, 2 min, 4 °C), after which the pellet was resuspended in 10 ml of culture medium. The cells were added to Percoll solution, mixed thoroughly, and centrifuged (50 × g, 4 °C). The viability of isolated hepatocytes, as estimated by trypan blue staining, was usually 80–90%. The cells were seeded onto collagen I-coated 12-well plates (50 µg/ml; Becton Dickinson) at 150,000 viable cells/well in Williams E medium (Lonza) supplemented with 10% FBS, 2 mM l-glutamine (Lonza), 1% penicillin–streptomycin (Lonza), 1 mM sodium pyruvate solution (Lonza), 1% ITS Liquid Media Supplement (Sigma), 7.5 µg/ml hydrocortisone (Sigma), and 20 ng/ml epidermal growth factor (Sigma), and the cells were maintained at 37 °C in a humidified atmosphere with 5% $CO_2$. After 5 h of attachment, the cultures were washed with phosphate-buffered saline and maintained in medium for the duration of the experiment.

### Statistical analysis

Student's *t* tests or Mann–Whitney *U* tests were used for statistical analysis of two groups. For more than two groups, ordinary two-way ANOVA was used. The number of animals or patient samples is indicated in the figure legends. All the results are presented as the mean ± SD. For graph preparation and statistical analysis, GraphPad Prism 10 (San Diego, CA, USA) was used. The *p* values are marked with asterisks in the charts (**p* <0.05, ***p* <0.01, ****p* <0.001 and *****p* <0.0001 *vs*. control).

The methods used for transduction, western blot analysis, mRNA extraction, real-time PCR analysis and staining are described in the supplementary materials and methods.

## Results

### MCPIP1 expression is downregulated in human HCC, and hepatocyte-specific *Zc3h12a* knockdown induces fibrosis and increases tumor growth *in vivo*

Previous research has demonstrated reduced levels of MCPIP1 in various tumors, including breast cancer,[15] clear cell

renal cell carcinoma (ccRCC),[16,17] and melanoma.[18] In this study, the role of MCPIP1 in HCC development was investigated. Analysis of HCC patient samples revealed a significant decrease in MCPIP1 expression in HCC tissue (Fig. 1A–D). Compared with adjacent non-tumor tissue samples, stage 1 and 2 HCC tissue samples presented significantly lower *ZC3H12A* expression (Fig. 1A–C). Across individual cancerous tissues, a decrease in the MCPIP1 level was observed in most samples regardless of tumor stage compared with non-cancerous tissues (Fig. 1A–C). Moreover, analysis of HCC patient databases revealed that the protein level of MCPIP1 decreased with HCC progression ($p$ = 5.70E-03) (Fig. 1D).

To determine whether the lack of MCPIP1 in hepatocytes and the resulting inflammation are involved in chemical hepatocarcinogenesis, we used a DEN-induced HCC model. For the present study, we generated an *in vivo* mouse model with liver-specific *Zc3h12a* gene knockout by crossing *Zc3h12a*$^{lox/lox}$ mice with liver-specific Cre-expressing *Alb*$^{Cre}$ transgenic mice (Fig. S1A). The mice were designated *Zc3h12a*$^{fl/fl}$*Alb*$^{Cre}$ mice (Fig. 1E).[19]

HCC predominantly affects men, and this sex discrepancy is also observed in mice exposed to the chemical carcinogen DEN. A single dose of DEN given to 2-week-old mice is sufficient to induce HCC, similar to human HCC, in 100% of male mice; however, females are largely resistant to carcinogenesis.[20] Surprisingly, a lack of MCPIP1 in female hepatocytes significantly induced tumorigenesis. Almost all the females in the *Zc3h12a*$^{fl/fl}$*Alb*$^{Cre}$ group developed tumors, whereas in the control group, only 2/15 females presented one small nodule (Fig. 1F). In the present study, all male mice developed tumors after 40 weeks of DEN administration. Compared with wild-type mice, *Zc3h12a*$^{fl/fl}$*Alb*$^{Cre}$ mice developed more tumors (Fig. 1G). These results indicated that MCPIP1 expression in the hepatocyte compartment is important for protection against HCC development, especially in female mice.

Masson's trichrome staining revealed collagen deposition, indicating fibrotic changes, with a more pronounced effect in *Zc3h12a*$^{fl/fl}$*Alb*$^{Cre}$ mice (Fig. 1H). Picrosirius red staining revealed the greatest degree of collagen deposition in hepatocytes from mice lacking MCPIP1 (Fig. 1I), suggesting enhanced fibrotic processes in addition to neoplastic changes.

*Zc3h12a* knockout mice that were not treated with DEN did not develop tumors (data not shown), and no macroscopic differences were observed between the sexes. However, a lack of MCPIP1 in hepatocytes induced intrahepatic bile duct pathology in the liver parenchyma and collagen deposition in *Zc3h12a*$^{fl/fl}$*Alb*$^{Cre}$ mice, as indicated by Masson's trichrome and Picrosirius red staining (Fig. S1C and D). Moreover, there was a slight increase in the influx of CD45-positive cells into the livers of *Zc3h12a*$^{fl/fl}$*Alb*$^{Cre}$ mice (Fig. S1E). Analysis of fibrosis-associated transcripts revealed that the absence of MCPIP1 significantly upregulated expression of *Ctgf,* a key mediator of tissue remodeling and fibrosis, as well as the expression of the mesenchymal markers *Vim* and *Fn1*. Additionally, *Mgl2* transcript levels were increased in *Zc3h12a*$^{fl/fl}$*Alb*$^{Cre}$ mice (Fig. S1F). Further, increased protein levels of Yes1, RhoA and Yap were detected in the livers of *Zc3h12a*$^{fl/fl}$*Alb*$^{Cre}$ mice (Fig. S1G). These results suggested that the fibrotic changes are induced by a lack of MCPIP1 expression in hepatocytes (Fig. S1).

**MCPIP1 liver knockout changes hepatocyte metabolism and increases fibrosis and inflammation**

Glutamine synthetase in the liver is expressed in a small, perivenous population of highly specialized hepatocytes, and it plays a key role in nitrogen metabolism and ammonia detoxification. The number of glutamine synthetase-positive cells was increased in the livers of *Zc3h12a* (which encodes MCPIP1) knockout mice and after DEN administration (Fig. 2A, B). In addition, the absence of *Zc3h12a* in hepatocytes increased the expression of a-SMA, which is positively correlated with the degree of fibrosis. a-SMA staining revealed enhanced fibrotic processes in female mice lacking MCPIP1 in hepatocytes (Fig. 2C). Immunofluorescence staining for the leukocyte marker CD45 and the monocyte marker CD68 revealed increased staining intensity in *Zc3h12a*$^{fl/fl}$*Alb*$^{Cre}$ mice (Fig. 2D,E), especially in areas with tumor cells (Fig. 2F), indicating the activation of the microenvironment in MCPIP1-deficient livers. The transcript levels of macrophage galactose-type C-type lectin 2 *(Mgl2*), *Cd3e* (T cell marker), and *Cd14* (monocyte/macrophage marker) were evaluated. Treatment of *Zc3h12a*$^{fl/fl}$*Alb*$^{Cre}$ mice with DEN resulted in the highest levels of *Mgl2* and *Cd14,* and *Zc3h12a*$^{fl/fl}$*Alb*$^{Cre}$ mice that did not receive DEN exhibited the highest transcript levels of *Cd3e* (Fig. 2G). Moreover, the expression of the proinflammatory markers *Tnfa*, *Ifng*, *Csf2* and *Casp1* was increased in *Zc3h12a*$^{fl/fl}$*Alb*$^{Cre}$ mice 12 and 24 weeks after DEN administration (Fig. 2H).

**MCPIP1 knockout leads to increased activation of the c-met and β-catenin signaling pathways and drives malignant HCC phenotypes by activating the STAT3/NFκB pathway in female mice**

Among the principal pathways involved in HCC development, the Wnt/β-catenin pathway, c-Met protooncogene activation, and EMT play prominent roles at 40 weeks after DEN administration, *Zc3h12a*$^{fl/fl}$*Alb*$^{Cre}$ mice exhibited stronger β-catenin and c-Met signals, indicating that a lack of MCPIP1 may enhance changes that lead to liver damage (Fig. 3A,B). In MCPIP1-deficient livers, immunohistochemical analyses revealed increased β-catenin activation in both the membrane and the cytoplasm of hepatocytes (Fig. 6A). Additionally, the expression of *Ctnnb1, Myc,* and *Mmp2* significantly increased*,* suggesting that fibrosis progression occurred in females (Fig. 2C,D).

Immunohistochemistry analysis of β-catenin revealed that a lack of MCPIP1 led to strong β-catenin activation, even in the absence of DEN (Fig. S2A). In mice that did not receive DEN, the lack of MCPIP1 in the livers of mice of both sexes led to a significant increase in the level and activation of c-Met (Fig. S2B–D). Western blot analysis confirmed that the level of the active c-Met receptor phosphorylated at Tyr1234/1235 increased (Fig. S2C and D). Furthermore, the loss of MCPIP1 led to increased transcript levels of the fibrosis and the inflammatory response markers *Mmp2, Il1b, Ctnnb1, Hgf, Cxcl12, Cxcr4, Zeb1* and *Twist* (Fig. S2E and F). Furthermore, females presented more pronounced differences (Fig. S2E and F).

MCPIP1 plays a pivotal role as a negative regulator of NF-κB, which is important for tumor promotion in inflammation-

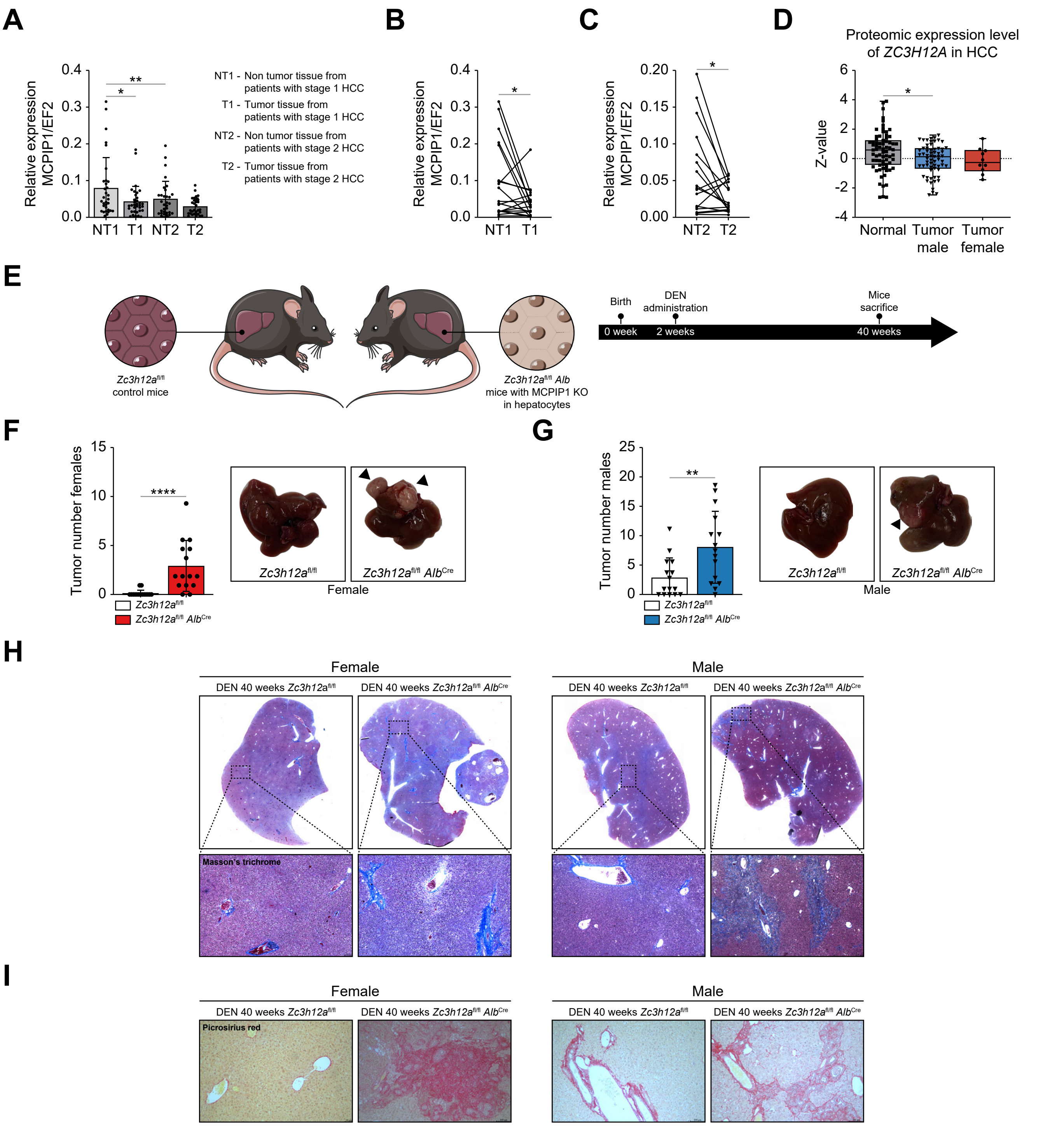


**Fig. 1. *ZC3H12A* expression decreases in tumor samples and promotes HCC development in mice after DEN administration.** (A) mRNA expression of MCPIP1 (*ZC3H12A*) in stage 1 and 2 HCC patient samples compared to non-tumor tissue, quantified with real-time PCR, *EF2* was used as the reference gene. Non-tumor stage 1 n = 32, non-tumor stage 2 n = 31, tumor stage 1 n = 34, tumor stage 2, n = 31. *p* values were estimated using one-way ANOVA, **p* <0.05. (B) Correlation between non-tumor stage 1 and tumor stage 1 samples, n = 19. (C) Correlation between non-tumor stage 2 and tumor stage 2 samples, n = 18. *p* values were estimated using unpaired Student's *t* test, **p* <0.05. (D) Analysis of HCC patient databases proteomic expression of *ZC3H12A* gene (*p* = 5.70E-03). (E) Schematic representation of the *in vivo* model for *Zc3h12a*$^{fl/fl}$*Alb*$^{Cre}$ mice with *Zc3h12a* gene knockout in hepatocytes and cholangiocytes and *Zc3h12a*$^{fl/fl}$ control mice. (F,G) Tumor number in *Zc3h12a*$^{fl/fl}$*Alb*$^{Cre}$ and *Zc3h12a*$^{fl/fl}$ mice n = 15, 40 weeks after DEN administration. The results are presented as the mean ± SD with dot plot. *p* values were estimated using unpaired Student's *t* test, ***p* <0.01, *****p* <0.0001. (H,I) Representative images of Masson's trichrome and Picrosirius red staining for *Zc3h12a*$^{fl/fl}$*Alb*$^{Cre}$ and *Zc3h12a*$^{fl/fl}$ mice, 40 weeks after DEN administration. DEN, diethylnitrosamine; HCC, hepatocellular carcinoma.

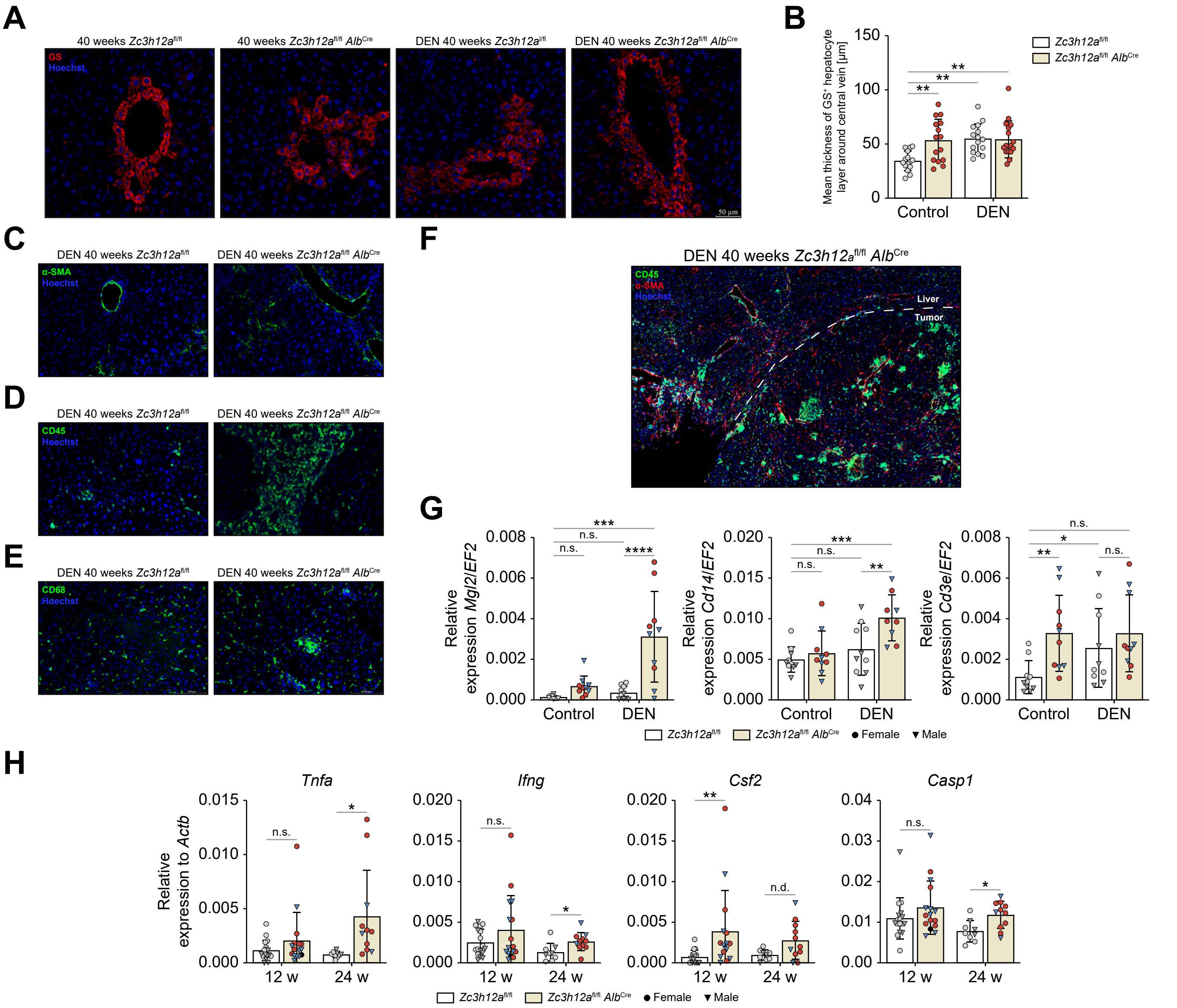


**Fig. 2. MCPIP1 liver knockout changes hepatocyte metabolism, increases fibrosis and inflammation.** (A) Representative images of glutamine synthetase immunofluorescent staining of liver *Zc3h12a*$^{fl/fl}$*Alb*$^{Cre}$ and *Zc3h12a*$^{fl/fl}$ female mice 40 weeks after DEN administration (Hoechst for nuclei). (B) Calculation of mean thickness of GS-positive hepatocytes layer around central vein. (C- E) Representative images of α-SMA, CD45 and CD68 immunofluorescent staining of liver *Zc3h12a*$^{fl/fl}$*Alb*$^{Cre}$ and *Zc3h12a*$^{fl/fl}$ female mice 40 weeks after DEN administration (Hoechst for nuclei). (F) Representative images of α-SMA and CD45 immunofluorescent staining of liver *Zc3h12a*$^{fl/fl}$*Alb*$^{Cre}$ and *Zc3h12a*$^{fl/fl}$ male mice (Hoechst for nuclei). (G) mRNA expression level of *Mgl2, Cd3e, Cd14. EF2* was used as the reference gene. Females; n = 5 per group, males; n = 4-5 per group. (H) mRNA expression level of *Tnfa, Ifng, Csf2, Casp1. Actb* was used as the reference gene. The results are presented as the mean ± SD with dot plot. *p* values were estimated using two-way ANOVA, $^*p < 0.05$, $^{**}p < 0.01$, $^{***}p < 0.001$, $^{****}p < 0.0001$. DEN, diethylnitrosamine; GS, glutamine synthetase.

associated liver cancer.[7,11] Compared with that in the control group, the total and active phosphorylated forms of NF-κB p65 in the female liver and the total NF-κB p65 protein level in the male liver significantly increased 40 weeks after DEN administration (Fig. 3E–H). Another key player in liver inflammation and tumorigenesis is signal transducer and activator of transcription 3 (STAT3), an oncogenic transcription factor that is critical for HCC development.[7] The levels and activation of STAT3 were increased in the livers of female mice with MCPIP1-deficient hepatocytes. Moreover, there were increased levels of p38 (T180/Y182) and ERK MAPK activation (Fig. 3E,F); however, these pathways were not activated in the livers of male mice (Fig. 3G,H). These results indicated that the development of HCC in female *Zc3h12a*$^{fl/fl}$*Alb*$^{Cre}$ mice is linked to the pronounced activation of the NF-κB and STAT3 signaling pathways, which are involved in inflammation-mediated hepatocarcinogenesis.[7]

## MCPIP1 deficiency activates transcriptomic changes that lead to HCC development

To investigate the importance of MCPIP1 at different stages of HCC development, we investigated DEN-induced hepatocarcinogenesis over time (Fig. 4A). Because MCPIP1

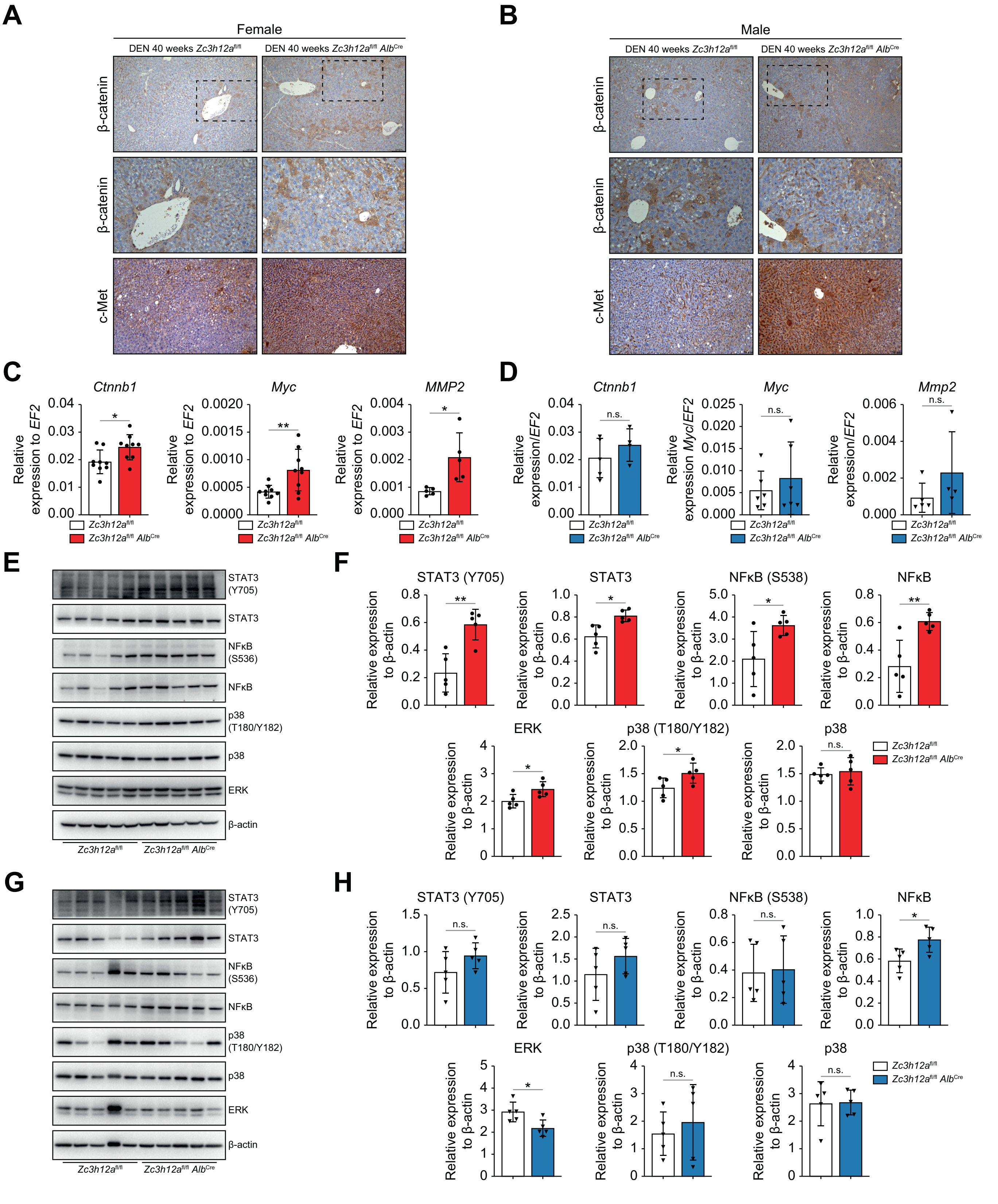


**Fig. 3. MCPIP1 knockout leads to the increased activation of the c-Met and β-catenin signaling pathways and drives HCC malignant phenotypes by activating the STAT3/NFκB pathway in female mice.** (A,B) Representative images of β-catenin and c-Met IHC staining of liver 42 weeks old *Zc3h12a*$^{fl/fl}$*Alb*$^{Cre}$ and *Zc3h12a*$^{fl/fl}$ mice males/females after DEN administration. (C,D) mRNA expression level of *Ctnnb1, Mmp2* and *Myc. EF2* was used as the reference gene. 42-weeks old females N = 5-9, males = 4-6 per group. (E-H) Western blot and densitometric analysis of STAT3, phospho-STAT3 (Y705), NF-κB, phospho-NF-κB (S536), p38 and phospho-p38 (T180/Y182), ERK protein level in *Zc3h12a*$^{fl/fl}$*Alb*$^{Cre}$ and *Zc3h12a*$^{fl/fl}$ female and male mice 40 weeks after DEN administration, n = 5 per group,

deficiency markedly affects HCC development in female mice, subsequent analyses focused on the livers of female animals. To identify transcriptional programs associated with a lack of MCPIP1, livers isolated from *Zc3h12a*$^{fl/fl}$*Alb*$^{Cre}$ mice (*Zc3h12a* gene knockout mice) and control mice after DEN administration were subjected to gene expression profiling via RNA sequencing (RNA-seq) and pathway analysis. The genes were dysregulated in the livers of *Zc3h12a*$^{fl/fl}$*Alb*$^{Cre}$ and control mice at 12 and 24 weeks after DEN administration (Fig. 4B). A total of 332 genes were upregulated and 341 were downregulated in the livers of *Zc3h12a*$^{fl/fl}$*Alb*$^{Cre}$ mice 12 weeks after DEN administration. Twenty-four weeks after DEN administration, 2,474 genes were upregulated and 1,593 were downregulated in livers lacking MCPIP1 compared with control livers (Fig. 4B). Twelve weeks after DEN administration, we assessed changes between wild-type and knockout mice (Figs 4C–F and S3). In *Zc3h12a*$^{fl/fl}$*Alb*$^{Cre}$ mice, there was significant enrichment of several cellular pathways, such as those related to KRAS signaling, angiogenesis, EMT and the inflammatory response (Figs 4F and S3). Global transcriptome analysis of liver samples 24 weeks after DEN administration revealed profound differences in gene expression between the livers of control and *Zc3h12a* knockout mice treated with DEN (Figs 4G–J and S3). A comparison of the tissues revealed hepatocyte MCPIP1-dependent effects on large sets of genes, mainly key genes for Wnt/β-catenin signaling, EMT, the inflammatory response, and IL6/JAK/STAT3 signaling (Figs 4J and S3). We subsequently analyzed changes in the expression of genes related to several pathways, such as liver fibrosis, oncogenes and EMT (Figs 4K and S3). Functional profiling and quantitative reverse-transcription PCR revealed that the transcript levels of factors associated primarily with fibrosis, EMT and cytokine-mediated signaling pathways, including *Spp1, Ctnnb1, Ctgf, Src, Zeb1, Vim, Tgfb2* and *Adam17*, were significantly increased in the livers of *Zc3h12a*$^{fl/fl}$*Alb*$^{Cre}$ female mice 12 weeks after DEN administration (Fig. 5A). Moreover, the active β-catenin levels were increased, and β-catenin translocated from the cell membrane to the cytoplasm (Fig. 5B). Analysis of liver samples collected 24 weeks after DEN administration revealed increased expression of multiple factors involved in the Wnt signaling pathway, including *Wnt4, Wnt5, Wnt6, Wnt7, Wnt11, Itpr3, Tbx3, Fzd8, Dvl2,* and *Axin* (Fig. 5C, D). MCPIP1 directly regulated the half-life of the *Il6* transcript, which activates the IL-6/JAK/STAT3 pathway. Liver samples collected 24 weeks after DEN administration exhibited elevated IL-6 secretion (Fig. 5E). Additionally, MCPIP1 deficiency increased the STAT3 levels in knockout mice, suggesting that a lack of MCPIP1 may be involved in amplifying destructive changes in hepatocytes after DEN administration (Fig. 5F).

### MCPIP1 knockout leads to the activation and nuclear translocation of β-catenin and the CREB1 transcription factor

To investigate the principal pathways involved in HCC development and to understand the mechanism underlying the observed phenotypic and genotypic changes in *Zc3h12a*$^{fl/fl}$*Alb*$^{Cre}$ mice, we analyzed primary hepatocytes isolated from 8-week-old *Zc3h12a*$^{fl/fl}$ and *Zc3h12a*$^{fl/fl}$*Alb*$^{Cre}$ mice. β-catenin activation involves a series of phosphorylation events that direct β-catenin to the nucleus. MCPIP1 deficiency in hepatocytes led to increased levels of the transcriptionally active form of β-catenin phosphorylated at S675 and S552 (Fig. 6A). In addition, active β-catenin (not phosphorylated at S45 but phosphorylated at S675) accumulated in the nuclei of hepatocytes (Fig. 6B). β-catenin phosphorylation at S675 induces the expression of *SPP1,* which encodes osteopontin, in hepatocytes, inducing their transdifferentiation to early hepatocyte progenitors and thus promoting a malignant cell fate.[21] Osteopontin induces β-catenin activation and contributes to maintaining the stem-like properties and tumorigenicity of hepatic progenitor cells in the liver.[22] In the present study, MCPIP1 deficiency and β-catenin S675 phosphorylation induced the expression of *Spp1* in hepatocytes (Fig. 6C).

IL-6 regulates the JAK/PI3K/Akt/CREB signaling pathway in hepatocytes.[23] In the present study, MCPIP1 deficiency increased the hepatic expression of *Creb1* and *Tgfb2* (Fig. 6C), and high levels of these factors were maintained in 12- and 24-week-old mice (Fig. 5A). Moreover, MCPIP1 deficiency in hepatocytes increased the level of its direct target, *Il6,* which directly affects hepatocyte dedifferentiation[24] (Fig. 6C). In addition, MCPIP1-deficient hepatocytes exhibited increased levels of protumorigenic *Adam17* (Fig. 6C), which regulates IL-6 trans-signaling.[25] Further, NF-kB, Akt, Erk, c-Met and STAT3 were phosphorylated in MCPIP1-defcient hepatocytes (Fig. 6D). Deletion of *Zc3h12a* induced the activation (phosphorylation at S133) and translocation of CREB1 into the cell nucleus (Fig. 6E–G).

Analysis of HCC patient databases from the CPTAC (Clinical Proteomic Tumor Analysis Consortium) and the ICPC (International Cancer Proteogenome Consortium) confirmed a notable increase in the protein expression of SPP1 ($p$ = 6.58E-14), CREB1 ($p$ = 1.10E-15), TGF-β2 ($p$ = 3.98E-11), CTGF ($p$ = 7.67E-15), and ADAM17 ($p$ = 1.19E-13) (Fig. 7A).[26] The gene expression levels of *SPP1* ($p$ = 1.90E-12), *CREB1* ($p$ <1E-12), *TGF-β2* ($p$ = 7.61E-05), *CTGF* ($p$ = 9.97E-01), *ADAM17* ($p$ <1E-12), and *MET* ($p$ = 1.32E-10) were also significantly increased (Fig. 7B).[26] Analysis of samples from patients with stage I and II HCC revealed higher *CTNNB1*, *TGFb2* and *SPP1* expression in tumors than in adjacent non-tumor tissue (Fig. 7C). These results may, at least partially, explain the protective role of MCPIP1 and the sex disparities associated with a higher level of MCPIP1 in females.

## Discussion

HCC is a complex multistep process that involves the early transformation of hepatocytes and the subsequent development of HCC. Although progress has been made, the pathophysiology of HCC remains incompletely understood, and most patients with HCC are not eligible for curative surgery because of the advanced stage of the disease at the time of diagnosis.

densitometric quantification with β-actin as the loading control. The results are presented as the mean ± SD with dot plot. $p$ values were estimated using unpaired Student's $t$ test or Mann-Whitney $U$ test, *$p$ <0.05, **$p$ <0.01. DEN, diethylnitrosamine; HCC, hepatocellular carcinoma; IHC, immunohistochemistry.

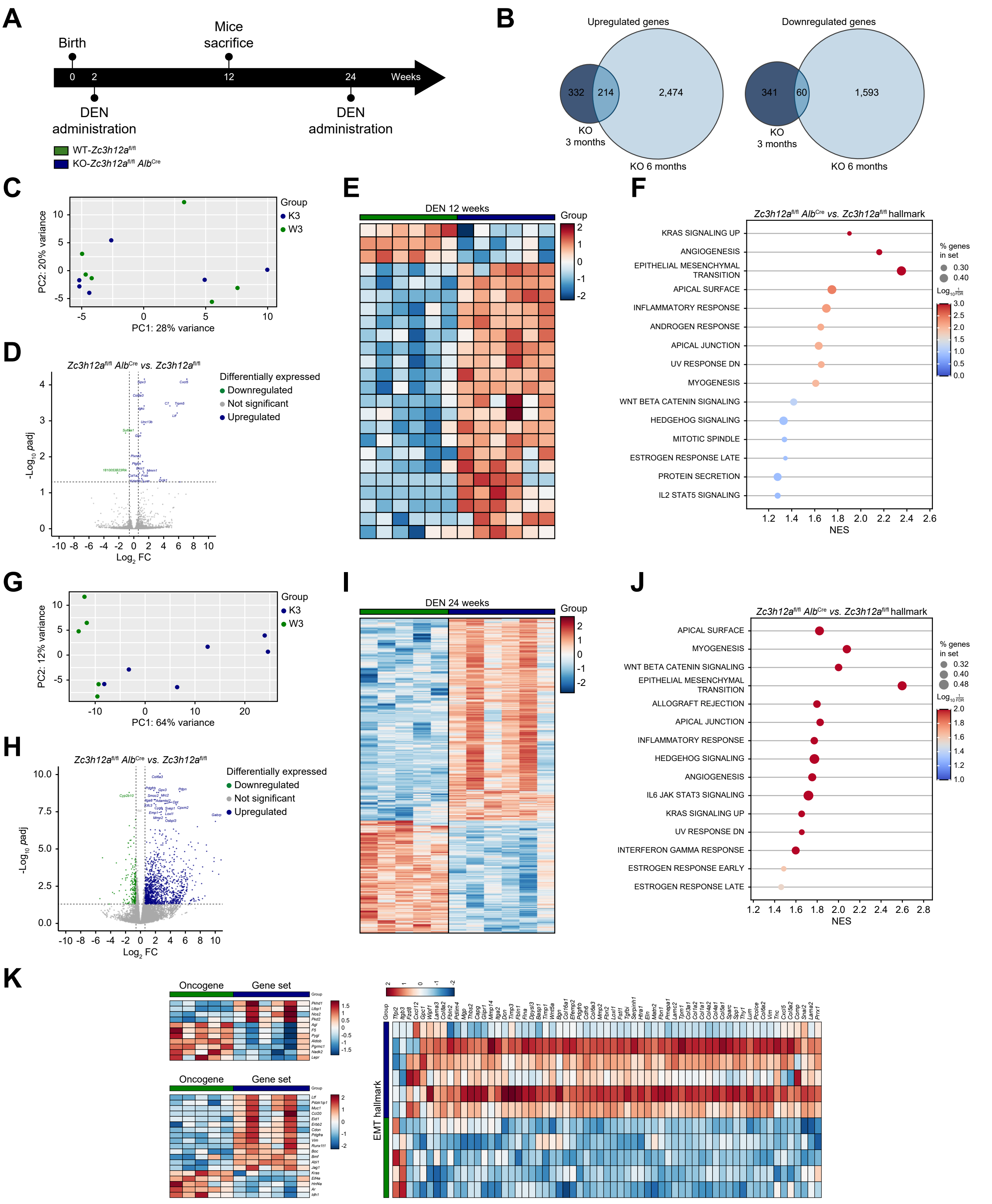


**Fig. 4. Lack of MCPIP1 activates transcriptomic changes leading to HCC development.** (A) Timelines for the study. (B) Diagram showing number of upregulated and downregulated genes in *Zc3h12a*$^{fl/fl}$*Alb*$^{Cre}$ female mice 12 and 24 weeks after DEN administration (*p* value <0.05). (C) PCA chart - comparison of *Zc3h12a*$^{fl/fl}$ control mice (green) and *Zc3h12a*$^{fl/fl}$*Alb*$^{Cre}$ mice (blue) 12 weeks after DEN administration. (D) Volcano plot shows the downregulated (green) and upregulated (blue) genes in *Zc3h12a*$^{fl/fl}$*Alb*$^{Cre}$ females compared to controls 12 weeks after DEN administration, *padj* ≤0.05. (E) Heatmap of the downregulated and upregulated genes

MCPIP1 has been reported to be a potential tumor suppressor in ccRCC that regulates tumor cell proliferation, tumor cell survival, tumor growth, vascularization and metastasis.[16] The present study revealed that MCPIP1 (*ZC3H12A*) expression was significantly lower in tumor tissue than in adjacent healthy liver tissue from patients with HCC, suggesting a potential regulatory role for MCPIP1 in hepatocarcinogenesis. However, larger public datasets do not consistently report significant reductions in *ZC3H12A* mRNA levels. This discrepancy may reflect the limitations of bulk RNA-seq in heterogeneous tumor samples, where signals from tumor cells can be diluted by surrounding stromal and immune components. Additionally, normalization methods commonly used in RNA-seq analyses may mask moderate but biologically relevant differences between tumor and non-tumor tissues. In contrast, the present quantitative reverse-transcription PCR approach allowed more sensitive detection of gene-specific changes in expression, supporting a potential tumor-suppressive role for MCPIP1 in liver cancer.

The present findings revealed that MCPIP1 deficiency in hepatocytes did not induce spontaneous tumor formation or a further increase in protumorigenic pathway activation in the livers of MCPIP1 knockout mice; instead, fibrotic changes were activated in the liver. The absence of MCPIP1 in hepatocytes increased YES1–YAP signaling, contributing to the downstream expression of profibrotic *Ctgf*, a key mediator of tissue remodeling and fibrosis. Additionally, the expression of EMT inducers (such as *Zeb1*, *Twist*, and *Ctnnb1*) and mesenchymal markers (such as *Fn1, Vim*, and *Mmp2*) increased, creating a profibrotic environment. Consequently, the liver tissue was fibrotic, with abundant collagen deposition. Hepatocyte-specific MCPIP1 knockout mice exhibited increased levels of the proinflammatory cytokines *Il1b* and *Il6*, which are directly regulated by MCPIP1,[13] as well as increased levels of *Hgf* and *Cxcl12*, which are involved in tumor growth and progression. Approximately 80% to 90% of HCC cases are associated with underlying cirrhosis, resulting from chronic liver inflammation, making cirrhosis a significant risk factor.[1] Furthermore, we have previously demonstrated reduced MCPIP1 protein levels in patients with MASLD, a condition associated with potential progression to end-stage liver disease, including HCC.[13]

Glutamine synthetase is an important liver enzyme responsible for catalyzing the ATP-dependent conversion of glutamate and ammonia to glutamine. Glutamine synthetase is localized primarily in perivenous hepatocytes and is a target of the Wnt/β-catenin pathway in the liver.[27] In MCPIP1-deficient livers, the expression and activity of glutamine synthetase were increased. This upregulation is often associated with early hepatocellular changes and is considered a marker of preneoplastic lesions, reflecting metabolic reprogramming and altered nitrogen handling.[28] Although the absence of MCPIP1 in the liver leads to pathological alterations, it is not sufficient to induce tumor development and must be accompanied by an additional oncogenic stimulus.

Sex dimorphism represents a prominent feature of HCC, with men being predominantly affected. This sex disparity is also observed in mice exposed to the DEN chemical carcinogen; DEN induces HCC in 100% of male mice, whereas female littermates are largely resistant to carcinogenesis.[4] Additionally, the administration of DEN leads to higher levels of circulating IL-6 in males than in females.[4] Unexpectedly, 40 weeks after DEN administration in the present study, almost all the female mice in the *Zc3h12a*$^{fl/fl}$*Alb*$^{Cre}$ group developed tumors. Although estrogens have been proposed to be general suppressors of HCC by reducing the proinflammatory effects of MyD88-mediated IL-6 secretion,[4] changes in the levels of estradiol, the expression of *Esr1*, or the expression of the *Foxa1*/*Foxa2* transcription factors that regulate sex hormones were not observed (data not shown). However, there were increased levels of IL-6 in *Zc3h12a*$^{fl/fl}$*Alb*$^{Cre}$ mice, which induced the compensatory proliferation of hepatocytes and the accumulation of DNA damage due to DEN.[4] The hepatocyte-specific MCPIP1 knockout model lacked the protective effect of MCPIP1, which is a key negative regulator of IL-6. MCPIP1 physically interacts with stem−loop structures in the 3′ untranslated region of *Il6* transcripts through its PIN domain, leading to mRNA destabilization and degradation.[29] These findings confirmed that increased levels of IL-6 play important roles in hepatocarcinogenesis and indicated that the level of MCPIP1 is important in protecting against HCC development in female mice.

The effects of MCPIP1 deficiency in hepatocytes may influence the hepatocyte phenotype and the microenvironment within the liver.[19] The influx of CD45+ and CD68+ immune cells was increased after DEN administration, suggesting macrophage activation. Moreover, the expression levels of the monocyte/macrophage markers *Cd14* and *Mgl2*, as well as the T cell marker *Cd3e,* were increased in the livers of *Zc3h12a* knockout mice after DEN administration. α-SMA staining revealed increased inflammation together with fibrosis, highlighting the importance of MCPIP1 in tissue microenvironment changes.

Early signaling events in HCC pathogenesis include the activation of Wnt/β-catenin signaling.[30] Studies have reported that mutation of *CTNNB1*, which encodes β-catenin, is among the key genetic events in human HCC.[31] Furthermore, Wnt/β-catenin has been implicated in HCC stemness, progression, metastasis, and drug resistance.[32,33] Up to 30–50% of HCC cases exhibit upregulated Wnt/β-catenin expression and *CTNNB1* gene mutations.[34] β-catenin plays dual roles, functioning as a component of the adherens junction complex at

in *Zc3h12a*$^{fl/fl}$*Alb*$^{Cre}$ mice (green) compared to *Zc3h12a*$^{fl/fl}$ control female mice (blue) 12 weeks after DEN administration, *p*adj ≤0.05. (F) Dotplot with significant enrichment in several cellular pathways in *Zc3h12a*$^{fl/fl}$*Alb*$^{Cre}$ female mice 12 weeks after DEN administration compared to control mice prepared in GSEApy Python package, FDR = 0,25. (G) PCA chart - comparison of *Zc3h12a*$^{fl/fl}$ control mice (green) and *Zc3h12a*$^{fl/fl}$*Alb*$^{Cre}$ mice (blue) 24 weeks after DEN administration. (H) Volcano plot shows the downregulated (green) and upregulated (blue) genes in *Zc3h12a*$^{fl/fl}$*Alb*$^{Cre}$ female mice compared to controls 24 weeks after DEN administration, *p*adj ≤0.05. (I) Heatmap of the downregulated and upregulated genes in *Zc3h12a*$^{fl/fl}$*Alb*$^{Cre}$ mice (green) compared to *Zc3h12a*$^{fl/fl}$ control female mice (blue) 24 weeks after DEN administration, *p*adj ≤0.05. (J) Dotplot with significant enrichment in several cellular pathways in *Zc3h12a*$^{fl/fl}$*Alb*$^{Cre}$ female mice 24 weeks after DEN administration compared to control mice prepared in GSEApy Python package, FDR = 0.25. (K) Heatmap of changed genes in several pathways - liver fibrosis, oncogene and EMT. DEN, diethylnitrosamine; EMT, epithelial-to-mesenchymal transition; FDR, false discovery rate; GSEApy, gene set enrichment analysis in Python; HCC, hepatocellular carcinoma; PCA, principal component analysis.

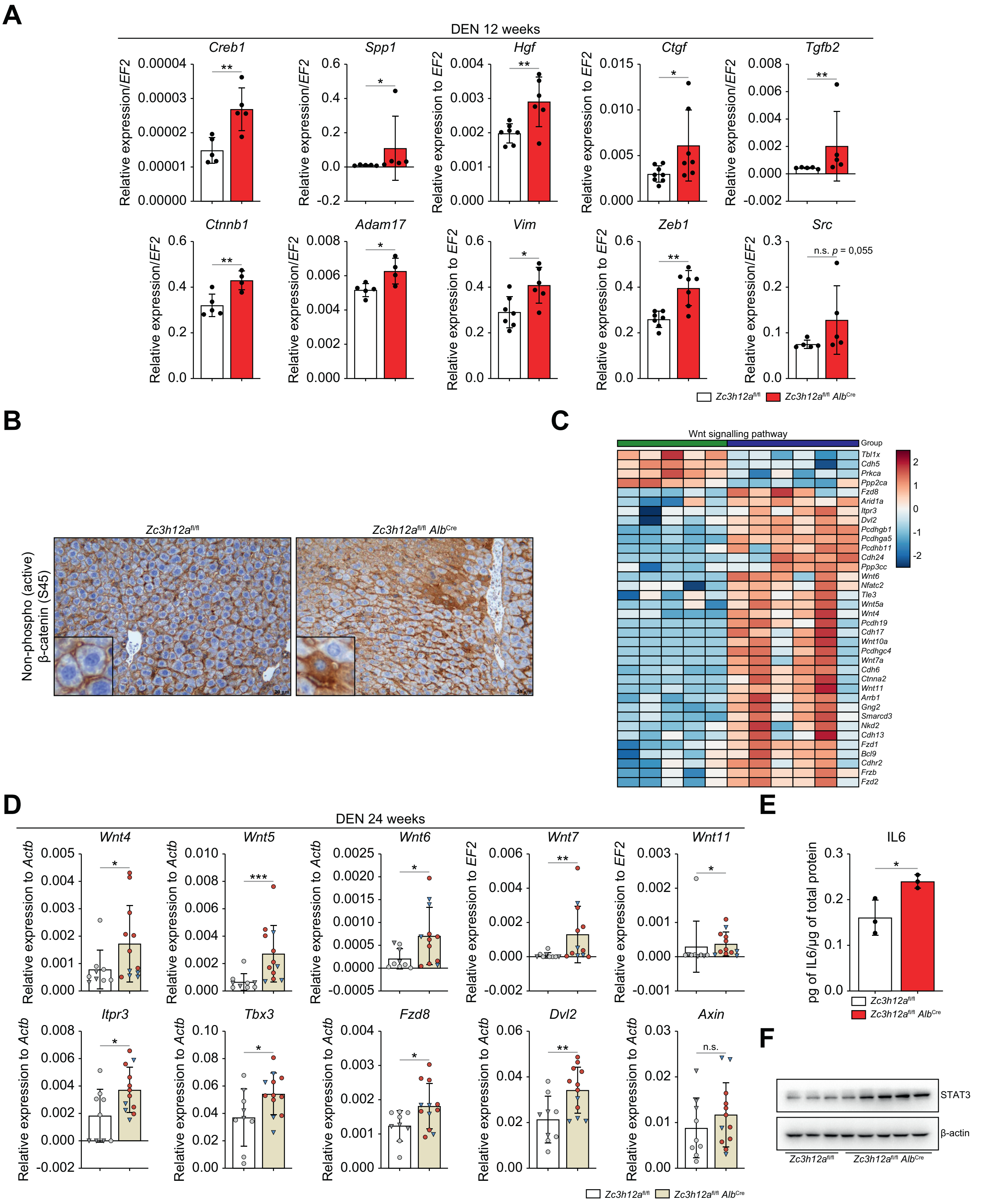


**Fig. 5.** ***Zc3h12a* knock out in the liver affects phenotype changes and IL-6/STAT signaling.** (A) mRNA expression level of *Creb1, Ctgf, Ctnnb1, Hgf, Spp1*, *Tgfb2*, *Vim, Zeb1, Adam17* and *Src. EF2* was used as the reference gene. 12-week-old females after DEN administration, n = 5 per group. (B) Representative images of β-catenin (S45) IHC staining of liver *Zc3h12a*$^{fl/fl}$*Alb*$^{Cre}$ and *Zc3h12a*$^{fl/fl}$ female mice 12 weeks after DEN administration. (C) Heatmap of changed genes in Wnt signaling pathway. (D) mRNA expression level of transcripts involved in Wnt signaling pathway in 24-weeks old females and males after DEN administration. *Actb* was used as

the membrane associated with cadherins and as a critical effector of the Wnt signaling pathway in the nucleus. Activation of β-catenin involves its phosphorylation at two sites, namely, S552 and S675, promoting its transcriptional activity as a cofactor for the TCF/LEF transcription factor.[35] However, the molecular mechanisms governing β-catenin activation and nuclear translocation remain largely unknown. Additionally, the transgenic expression of degradation-resistant β-catenin in the liver is insufficient to drive HCC initiation *in vivo*.[36,37] Hence, whether β-catenin activation is a driving mechanism or a cooperative event that supports HCC progression initiated by other oncogenic factors remains unclear.[38] β-catenin is a male-biased HCC driver whose mutation frequency is relatively high in HCC specimens from males. The present study revealed a notable increase in the level of the active form of β-catenin in female mice lacking MCPIP1 in hepatocytes, even shortly after DEN administration. Furthermore, active β-catenin translocated into the hepatocyte nucleus. Previous findings have demonstrated that MCPIP1 regulates the level, localization, and activity of β-catenin in ccRCC cells by modulating the expression of negative regulators of the Wnt pathway. The absence of MCPIP1 increases the transcriptionally active form of β-catenin, influencing the acquisition of mesenchymal features.[17] The present data indicated that in addition to β-catenin activation, the expression of other components of the Wnt signaling pathway was concomitantly upregulated in hepatocytes lacking MCPIP1. In the present study, inflammation-driven Wnt signaling activated β-catenin in hepatocytes, promoting proliferation and oncogenic transformation, as shown in liver injury and cancer models.[39] We hypothesize that MCPIP1, through its involvement in β-catenin and Wnt signaling activation, may contribute to HCC initiation in female mice.

The present results indicate that the development of HCC in female *Zc3h12a*$^{fl/fl}$ *Alb*$^{Cre}$ mice was linked to the pronounced activation of the NF-κB and STAT3 signaling pathways, which are involved in inflammation-mediated hepatocarcinogenesis.[7] Increased levels of IL-6 and increased STAT3 activity have been reported in patients with HCC.[40] STAT3 may increase the nuclear localization of β-catenin in colorectal cancer through potential crosstalk between the Wnt/β-catenin pathway and the IL6/gp130/STAT3 pathway.[41] Thus, we hypothesized that the regulation of IL-6 levels and the activation of β-catenin by MCPIP1, as in the case of ccRCC,[17] may be missing. Another activator of β-catenin in hepatocytes lacking MCPIP1 may be osteopontin, which is encoded by *SPP1* and promotes hepatic progenitor cell expansion and tumorigenicity.[22] In the present study, the expression of *Spp1* was increased in mice lacking MCPIP1 and in patients with HCC.

IL-6 also activates the JAK/PI3K/Akt/CREB signaling pathway in hepatocytes.[23] CREB1 belongs to the CREB/activating transcription factor family of transcription factors and is activated by S133 phosphorylation, which is mediated by AKT. CREB factors promote tumorigenesis in many cancers. Patients with HCC and increased expression and phosphorylation of CREB1 at S133 have decreased overall survival and a greater risk of tumor relapse.[42] The present study revealed that a lack of MCPIP1 in hepatocytes increased CREB1 expression and activation, *i.e*. S133 phosphorylation. CREB1 binds to the identified CRE in the proximal region of the *TGFB2* promoter. The present results revealed increased *TGFB2* expression in both mice lacking MCPIP1 and patients with HCC. In support of their importance in cancer biology, *CREB1* and *TGFB2* mRNA levels are positively correlated in human glioblastoma samples.[43] Moreover, recent evidence has indicated that phosphorylated CREB1 (S133) binds to a *Ctnnb1* enhancer and regulates β-catenin transcription. In the present MCPIP1-deficient hepatocyte model, CREB1 activation served as a significant mediator of HCC development through its influence on β-catenin expression.[44]

Fate-tracing studies have demonstrated that HCC originates primarily from hepatocytes. In the initial stages of HCC, transformed hepatocytes, regardless of their proliferation status, promote the expansion of hepatic progenitor cells. However, the precise mechanisms initiated by injured hepatocytes in this process remain unclear.[45] The present study highlighted the crucial role of MCPIP1 in hepatocytes, preserving liver homeostasis, preventing fibrosis development, and acting as a potent suppressor of liver tumor development, particularly in female mice resistant to tumorigenesis, revealing the importance of MCPIP1 as a guardian/cell protector. While the present research highlighted the role of MCPIP1 in tumor biology and the intricate regulation of the development of HCC, the full extent of the actions of MCPIP1 and the complexities involved remain poorly understood.

A limitation of the present study was the use of the *Alb*-cre transgenic line for *Zc3h12a* deletion. *Alb-cre* becomes active around embryonic Day 18, a time when hepatoblasts are still bipotential and capable of giving rise to both hepatocytes and cholangiocytes. Although the *Alb* promoter drives predominantly hepatocyte-specific recombination postnatally, recombination during late embryogenesis may influence both lineages, especially under pathological conditions where cell plasticity and transdifferentiation may occur. This is particularly relevant considering the ductular reaction and biliary alterations observed in the present model. Moreover, differences between constitutive (*Alb*-cre) and inducible (AAV8-*Tbg*-Cre) models have been previously noted, including in *Ctnnb1* knockout mice, in which early deletion induces compensatory mechanisms that alter adult phenotypes.[46] These factors should be considered when interpreting the phenotypic spectrum observed in the present study, as they may partially explain some of the biliary features and suggest a developmental component to the observed pathogenesis. While acknowledging the limitations associated with the developmental timing of *Alb*-cre, the present model also offers several important strengths. Chronic, hepatocyte-targeted deletion of *Zc3h12a* allows investigation of liver tumorigenesis in the

the reference gene. (E) Analysis of IL-6 protein level in *Zc3h12a*$^{fl/fl}$*Alb*$^{Cre}$ and *Zc3h12a*$^{fl/fl}$ female mice 24 weeks after DEN administration, n = 3 per group. (F) Analysis of STAT3 in *Zc3h12a*$^{fl/fl}$*Alb*$^{Cre}$ and *Zc3h12a*$^{fl/fl}$ female mice 24 weeks after DEN administration. The results are presented as the mean ± SD with dot plot. *P* values were estimated using unpaired Student's *t* test or Mann-Whitney *U* test, **p* <0.05, ***p* <0.01, ****p* <0.001. DEN, diethylnitrosamine; HCC, hepatocellular carcinoma; IHC, immunohistochemistry.

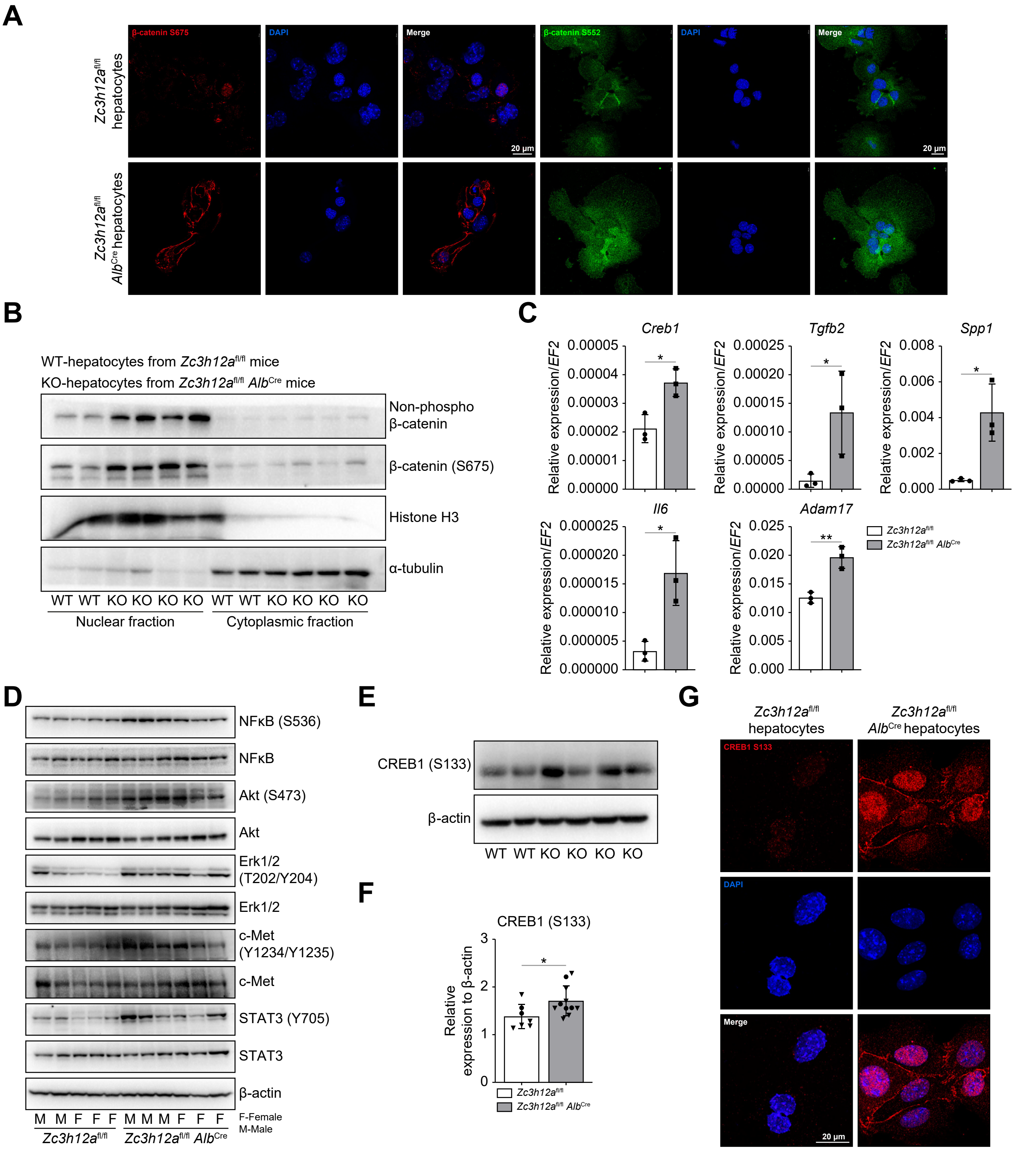


**Fig. 6. CREB1 as key player in liver changes after *Zc3h12a* knockout.** (A) Confocal staining of β-catenin phosphorylated on S552 and S675 in hepatocytes of females *Zc3h12a*$^{fl/fl}$*Alb*$^{Cre}$ mice and *Zc3h12a*$^{fl/fl}$ control mice (DAPI for nuclei). (B) Analysis of β-catenin (S675) and non-phosphorylated (S45) β-catenin protein level in cytoplasmic and nuclear fraction in hepatocytes of *Zc3h12a*$^{fl/fl}$*Alb*$^{Cre}$ mice and *Zc3h12a*$^{fl/fl}$ control mice. Histon H3 was used as nuclear loading control and α-tubulin was used as cytoplasmic loading control. (C) mRNA expression level of *Creb1*, *Tgfb2*, *Spp1*, *Adam17* and *Il-6*. *EF2* was used as the reference gene. 8-week-old females, n = 3 per group. (D) Analysis of NF-kB, phospho-NF-kB (S536), Akt, phospho-Akt (S473), ERK1/2, phospho-ERK (T202/Y204), c-Met, phospho-c-Met (Y1234/1235), STAT3, phospho-STAT3 (Y705), with β-actin as the loading control in *Zc3h12a*$^{fl/fl}$*Alb*$^{Cre}$ and *Zc3h12a*$^{fl/fl}$ hepatocytes isolated from females and males. (E,F) Analysis of CREB1 (S133) protein level in *Zc3h12a*$^{fl/fl}$*Alb*$^{Cre}$ and *Zc3h12a*$^{fl/fl}$ hepatocytes isolated from females and males (indicated on the graph), n = 2-4 per group, densitometric quantification with β-actin as the loading control. (G) Confocal staining of Creb1 phosphorylated on Ser133 in hepatocytes of *Zc3h12a*$^{fl/fl}$*Alb*$^{Cre}$ and *Zc3h12a*$^{fl/fl}$ control mice (DAPI for nuclei). The results are presented as the mean ± SD with dot plot. *P* values were estimated using unpaired Student's *t* test, Mann-Whitney *U test*, *$p$ <0.05, **$p$ <0.01, ***$p$ <0.001, ****$p$ <0.0001.

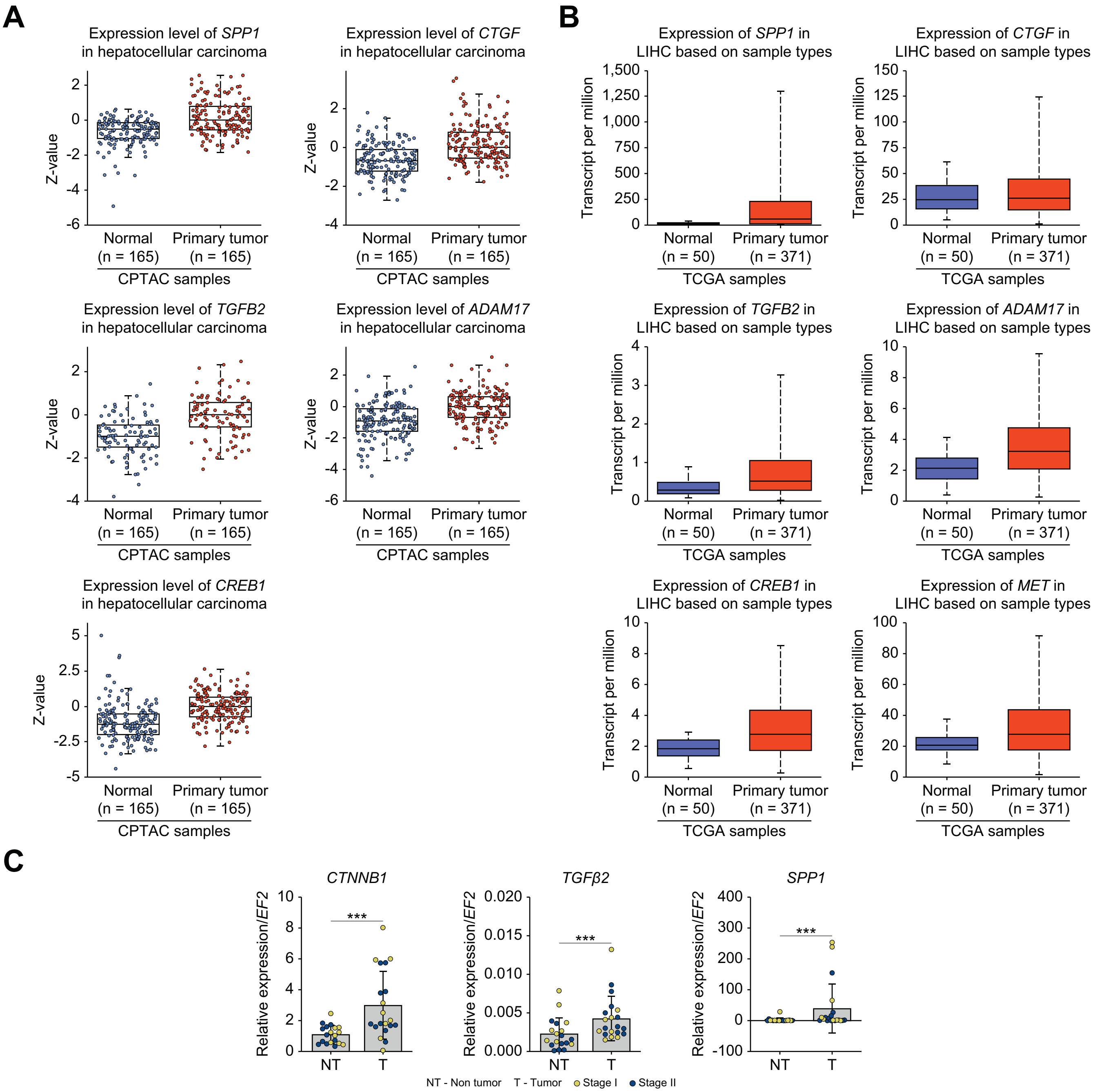


**Fig. 7. *CTNNB1* expression is lower in tumor samples compared to adjacent non tumor samples.** (A) Protein expression analysis option using data from the CPTAC and the ICPC datasets - https://ualcan.path.uab.edu/analysis-prot.html. *SPP1* (*p* = 6.58E-14), *CREB1* (*p* = 1.10E-15), *TGFβ2* (*p* = 3.98E-11), *CTGF* (*p* = 7.67E-15) and *ADAM17* (*p* = 1.19E-13). Normal n = 165, Primary tumor n = 165. Z-values represent standard deviations from the median across samples for the given cancer type. Log2 Spectral count ratio values from CPTAC were first normalized within each sample profile, then normalized across samples. (B) Expression analysis using data from TCGA. *SPP1* (*p* = 1.90E-12), *CREB1* (*p* <1E-12), *TGFβ2* (*p* = 7.61E-05), *CTGF* (*p* = 9.97E-01), *ADAM17* (*p* <1E -12) and *MET* (*p* = 1.32E-10). Normal n = 50, Primary tumor n = 371. (C) mRNA expression level of *CTNNB1, TGFB2* and *SPP1* in tumor (T) and non-tumor (NT) samples (n = 20 per group; Stage 1 n = 11; Stage 2 n = 9). *EF2* was used as the reference gene. The results are presented as the mean ± SD with dot plot. *P* values were estimated using unpaired Student's *t* test, ***p* <0.01, ****p* <0.001. CPTAC, Clinical Proteomic Tumor Analysis Consortium; ICPC, International Cancer Proteogenome Consortium; TCGA, The Cancer Genome Atlas.

context of progressive inflammation and fibrosis, closely mimicking many clinical cases of HCC. Finally, the present transcriptomic analysis was performed on female livers, in which the inflammatory and tumor phenotypes were most robust. While male MCPIP1-deficient mice were included in other parts of the study, a direct male *vs*. female transcriptomic comparison remains an important next step to better understand sex-specific disease mechanisms.

## Affiliations

[1]Department of General Biochemistry, Faculty of Biochemistry, Biophysics and Biotechnology, Jagiellonian University, Krakow, Poland; [2]Doctoral School of Exact and Natural Sciences, Jagiellonian University, Lojasiewicza 11, 30-348, Kraków, Poland; [3]TGF-$\beta$ and Cancer Group, Oncobell Program, Bellvitge Biomedical Research Institute (IDIBELL), Gran Via de l'Hospitalet, 199, 08908 Barcelona, Spain; [4]CIBEREHD, National Biomedical Research Institute on Liver and Gastrointestinal Diseases, Instituto de Salud Carlos III, Spain; [5]Unit of Bioinformatics for Precision Oncology, Institut Català d'Oncologia (ICO), L'Hospitalet de Llobregat, Barcelona, Spain; [6]Preclinical and Experimental Research in Thoracic Tumors (PReTT), Oncobell Program, Bellvitge Biomedical Research Institute (IDIBELL), L'Hospitalet de Llobregat, Barcelona, Spain; [7]Department of Pathomorphology, Faculty of Medicine, Jagiellonian University Medical College, Krakow, Grzegorzecka 16, 31-531, Poland; [8]Department of Surgery, Liver Transplant Unit, University Hospital of Bellvitge and Faculty of Medicine and Health Sciences, University of Barcelona, L'Hospitalet de Llobregat, Barcelona, Spain


## Abbreviations

ccRCC, clear cell renal cell carcinoma; CREB1, cAMP-responsive element-binding protein 1; DEN, diethylnitrosamine; EMT, epithelial-to-mesenchymal transition; HCC, hepatocellular carcinoma; IL-6, interleukin 6; MASLD, metabolic dysfunction-associated steatotic liver disease; MCPIP1, monocyte chemoattractant protein-induced protein 1; Mgl2, macrophage galactose C-type lectin 2; RNA-seq, RNA sequencing; STAT3, signal transducer and activator of transcription 3.


## Acknowledgment and financial support

This work was supported in part by research grants from National Science Centre no. 2017/26/E/NZ5/00691 and 2022/45/B/NZ5/01973 to K.M., 2021/41/N/NZ4/04187 to O.K., 2022/47/B/NZ5/02724 to P.M. and by research task under IDUJA – funded from the scientific subsidy of the Faculty of Biochemistry, Biophysics and Biotechnology, Jagiellonian University no. 19000082_N_25_47 to J.G. Part of the optical microscopy experiments: confocal imaging of the β-catenin and CREB presented in the Figure 6A and G were performed at the Bioimaging Laboratory which serves as an imaging core facility at the Faculty of Biochemistry, Biophysics and Biotechnology JU. We would like to thank Beata Rysiewicz, PhD from the Bioimaging Laboratory for her technical support.


## Conflict of interest

The authors declare no potential conflicts of interest.

Please refer to the accompanying ICMJE disclosure forms for further details.

## Authors' contributions

Conceptualization and design: OK, PM, KM; Formal Analysis: OK, PM, JG, RM, KM; Funding Acquisition: OK, PM, KM, JG; Investigation: PM, OK, RM, JG, E G-S, EB, JK, MG, AA, NP, MK, ER, KM; Methodology: OK, PM, JG, RM, KM; Analysis: OK, PM, JG, RM, IF, KM; Project Administration: OK, PM, KM; Data interpretation: OK, PM, IF, KM; Supervision: KM; Validation: OK, PM, RM, KM; Visualization: OK, PM, JG, RM; Writing: OK, PM, KM; All authors read and approved the final manuscript.

## Data availability

The authors declare that all the data supporting the findings of this study are available within the paper in the main text or the Supplementary Materials. Raw and processed data from next-generation sequencing were deposited at https://www.ncbi.nlm.nih.gov/bioproject/1198596 (accession number: PRJNA1198596).

## Supplementary data

Supplementary data to this article can be found online at https://doi.org/10.1016/j.jhepr.2026.101755.

## References

*Author names in bold designate shared co-first authorship.*


[1] Llovet JM, Kelley RK, Villanueva A, et al. Hepatocellular carcinoma. Nat Rev Dis Primers 2021;7(1):6. https://doi.org/10.1038/s41572-020-00240-3.

[2] Villanueva A. Hepatocellular carcinoma. Longo DL, editor. N Engl J Med 2019;380(15):1450–1462. https://doi.org/10.1056/NEJMRA1713263.

[3] Llovet JM, Ricci S, Mazzaferro V, et al. Sorafenib in advanced hepatocellular carcinoma. N Engl J Med 2008;359(4):378–390. https://doi.org/10.1056/NEJMOA0708857.

[4] Naugler WE, Sakurai T, Kim S, et al. Gender disparity in liver cancer due to sex differences in MyD88-dependent IL-6 production. Science 2007;317(5834):121–124. https://doi.org/10.1126/SCIENCE.1140485.

[5] Maeda S, Kamata H, Luo JL, et al. IKKbeta couples hepatocyte death to cytokine-driven compensatory proliferation that promotes chemical hepatocarcinogenesis. Cell 2005;121(7):977–990. https://doi.org/10.1016/J.CELL.2005.04.014.

[6] Sakurai T, He G, Matsuzawa A, et al. Hepatocyte necrosis induced by oxidative stress and IL-1 alpha release mediate carcinogen-induced compensatory proliferation and liver tumorigenesis. Cancer Cell 2008;14(2):156–165. https://doi.org/10.1016/J.CCR.2008.06.016.

[7] He G, Karin M. NF-κB and STAT3 - key players in liver inflammation and cancer. Cell Res 2011;21(1):159–168. https://doi.org/10.1038/CR.2010.183.

[8] Pikarsky E, Porat RM, Stein I, et al. NF-kappaB functions as a tumour promoter in inflammation-associated cancer. Nature 2004;431(7007):461–466. https://doi.org/10.1038/NATURE02924.

[9] **Zhang C**, **Zhou Y**, **Hu M**, et al. PLOD1 promotes the malignancy of hepatocellular carcinoma by facilitating the NF-κB/IL-6/STAT3-dependent TCA cycle. JHEP Rep 2025;7(5):101329. https://doi.org/10.1016/J.JHEPR.2025.101329/ATTACHMENT/F68C3941-CE6E-4A46-96D5-E366823F684E/MMC.

[10] Miekus K, Kotlinowski J, Lichawska-Cieslar A, et al. Activity of MCPIP1 RNase in tumor associated processes. J Exp Clin Cancer Res 2019;38(1):421. https://doi.org/10.1186/s13046-019-1430-6.

[11] Liang J, Saad Y, Lei T, et al. MCP-induced protein 1 deubiquitinates TRAF proteins and negatively regulates JNK and NF-kappaB signaling. J Exp Med 2010;207(13):2959–2973. https://doi.org/10.1084/jem.20092641.

[12] Pydyn N, Ferenc A, Trzos K, et al. MCPIP1 inhibits hepatic stellate cell activation in autocrine and paracrine manners, preventing liver fibrosis. Cell Mol Gastroenterol Hepatol 2024;17(6):887–906. https://doi.org/10.1016/j.jcmgh.2024.01.021.

[13] Pydyn N, Kadluczka J, Major P, et al. Hepatic MCPIP1 protein levels are reduced in NAFLD patients and are predominantly expressed in cholangiocytes and liver endothelium. Hepatol Commun 2023;7(3). https://doi.org/10.1097/HC9.0000000000000008. e0008-e0008.

[14] Lee SML, Schelcher C, Demmel M, et al. Isolation of human hepatocytes by a two-step collagenase perfusion procedure. J Vis Exp 2013;79:50615. https://doi.org/10.3791/50615.

[15] Lu W, Ning H, Gu L, et al. MCPIP1 selectively destabilizes transcripts associated with an antiapoptotic gene expression program in breast cancer cells that can elicit complete tumor regression. Cancer Res 2016;76(6):1429–1440. https://doi.org/10.1158/0008-5472.CAN-15-1115.

[16] Marona P, Gorka J, Mazurek Z, et al. MCPIP1 downregulation in clear cell renal cell carcinoma promotes vascularization and metastatic progression. Cancer Res 2017;77(18). https://doi.org/10.1158/0008-5472.CAN-16-3190.

[17] Gorka J, Marona P, Kwapisz O, et al. MCPIP1 inhibits Wnt/β-catenin signaling pathway activity and modulates epithelial-mesenchymal transition during clear cell renal cell carcinoma progression by targeting miRNAs. Oncogene 2021;40(50):6720–6735. https://doi.org/10.1038/s41388-021-02062-3.

[18] Szukala W, Lichawska-Cieslar A, Pietrzycka R, et al. Loss of epidermal MCPIP1 is associated with aggressive squamous cell carcinoma. J Exp Clin Cancer Res 2021;40(1). https://doi.org/10.1186/S13046-021-02202-3.

[19] Kotlinowski J, Hutsch T, Czyzynska-Cichon I, et al. Deletion of Mcpip1 in Mcpip1fl/flAlbCre mice recapitulates the phenotype of human primary biliary cholangitis. Biochim Biophys Acta Mol Basis Dis 2021;1867(5). https://doi.org/10.1016/J.BBADIS.2021.166086.

[20] Verna L, Whysner J, Williams GM. N-nitrosodiethylamine mechanistic data and risk assessment: bioactivation, DNA-adduct formation, mutagenicity, and tumor initiation. Pharmacol Ther 1996;71(1–2):57–81. https://doi.org/10.1016/0163-7258(96)00062-9.

[21] Zulehner G, Mikula M, Schneller D, et al. Nuclear beta-catenin induces an early liver progenitor phenotype in hepatocellular carcinoma and promotes tumor recurrence. Am J Pathol 2010;176(1):472–481. https://doi.org/10.2353/AJPATH.2010.090300.
[22] Liu Y, Cao L, Chen R, et al. Osteopontin promotes hepatic progenitor cell expansion and tumorigenicity via activation of β-catenin in mice. Stem Cells 2015;33(12):3569–3580. https://doi.org/10.1002/STEM.2072.
[23] Chou CH, Lai SL, Chen CN, et al. IL-6 regulates Mcl-1L expression through the JAK/PI3K/Akt/CREB signaling pathway in hepatocytes: implication of an anti-apoptotic role during liver regeneration. PLoS One 2013;8(6). https://doi.org/10.1371/JOURNAL.PONE.0066268.
[24] Li L, Cui L, Lin P, et al. Kupffer-cell-derived IL-6 is repurposed for hepatocyte dedifferentiation via activating progenitor genes from injury-specific enhancers. Cell Stem Cell 2023;30(3):283–299.e9. https://doi.org/10.1016/J.STEM.2023.01.009.
[25] Schumacher N, Rose-John S. ADAM17 orchestrates Interleukin-6, TNFα and EGF-R signaling in inflammation and cancer. Biochim Biophys Acta (BBA) - Mol Cell Res 2022;1869(1):119141. https://doi.org/10.1016/j.bbamcr.2021.119141.
[26] Chandrashekar DS, Karthikeyan SK, Korla PK, et al. UALCAN: an update to the integrated cancer data analysis platform. Neoplasia 2022;25:18–27. https://doi.org/10.1016/j.neo.2022.01.001.
[27] Cadoret A, Ovejero C, Terris B, et al. New targets of β-catenin signaling in the liver are involved in the glutamine metabolism. Oncogene 2002;21(54):8293–8301. https://doi.org/10.1038/sj.onc.1206118.
[28] **Kurosaki S**, **Nakagawa H**, **Hayata Y**, et al. Cell fate analysis of zone 3 hepatocytes in liver injury and tumorigenesis. JHEP Rep 2021;3(4). https://doi.org/10.1016/j.jhepr.2021.100315.
[29] Matsushita K, Takeuchi O, Standley DM, et al. *Zc3h12a* is an RNase essential for controlling immune responses by regulating mRNA decay. Nature 2009;458(7242):1185–1190. https://doi.org/10.1038/nature07924.
[30] Suzuki T, Yano H, Nakashima Y, et al. Beta-catenin expression in hepatocellular carcinoma: a possible participation of beta-catenin in the dedifferentiation process. J Gastroenterol Hepatol 2002;17(9):994–1000. https://doi.org/10.1046/J.1440-1746.2002.02774.X.
[31] **Torrecilla S**, **Sia D**, **Harrington AN**, et al. Trunk mutational events present minimal intra- and inter-tumoral heterogeneity in hepatocellular carcinoma. J Hepatol 2017;67(6):1222–1231. https://doi.org/10.1016/J.JHEP.2017.08.013.
[32] Yamashita T, Budhu A, Forgues M, et al. Activation of hepatic stem cell marker EpCAM by Wnt-beta-catenin signaling in hepatocellular carcinoma. Cancer Res 2007;67(22):10831–10839. https://doi.org/10.1158/0008-5472.CAN-07-0908.
[33] **Liu W**, **Liu Y**, **Liu H**, et al. Snail predicts recurrence and survival of patients with localized clear cell renal cell carcinoma after surgical resection. Urol Oncol 2015;33(2). https://doi.org/10.1016/j.urolonc.2014.08.003. 69.e1-10.
[34] **Schulze K**, **Imbeaud S**, **Letouzé E**, et al. Exome sequencing of hepatocellular carcinomas identifies new mutational signatures and potential therapeutic targets. Nat Genet 2015;47(5):505–511. https://doi.org/10.1038/NG.3252.
[35] Taurin S, Sandbo N, Qin Y, et al. Phosphorylation of β-catenin by cyclic AMP-dependent protein kinase. J Biol Chem 2006;281(15):9971–9976. https://doi.org/10.1074/jbc.M508778200.
[36] **Nejak-Bowen KN**, **Thompson MD**, Singh S, et al. Accelerated liver regeneration and hepatocarcinogenesis in mice overexpressing serine-45 mutant beta-catenin. Hepatology 2010;51(5):1603–1613. https://doi.org/10.1002/HEP.23538.
[37] Parsons MJ, Tammela T, Dow LE. WNT as a driver and dependency in cancer. Cancer Discov 2021;11(10):2413–2429. https://doi.org/10.1158/2159-8290.CD-21-0190.
[38] Kim E, Lisby A, Ma C, et al. Promotion of growth factor signaling as a critical function of β-catenin during HCC progression. Nat Commun 2019;10(1). https://doi.org/10.1038/S41467-019-09780-Z.
[39] Debebe A, Medina V, Chen CY, et al. Wnt/β-catenin activation and macrophage induction during liver cancer development following steatosis. Oncogene 2017;36(43):6020–6029. https://doi.org/10.1038/ONC.2017.207.
[40] Aleksandrova K, Boeing H, Nöthlings U, et al. Inflammatory and metabolic biomarkers and risk of liver and biliary tract cancer. Hepatology 2014;60(3):858–871. https://doi.org/10.1002/HEP.27016.
[41] Bollrath J, Phesse TJ, von Burstin VA, et al. gp130-mediated Stat3 activation in enterocytes regulates cell survival and cell-cycle progression during colitis-associated tumorigenesis. Cancer Cell 2009;15(2):91–102. https://doi.org/10.1016/J.CCR.2009.01.002.
[42] Steven A, Friedrich M, Jank P, et al. What turns CREB on? And off? And why does it matter? Cell Mol Life Sci 2020;77(20):4049–4067. https://doi.org/10.1007/S00018-020-03525-8.
[43] Rodón L, Gonzàlez-Juncà A, Del Mar Inda M, et al. Active CREB1 promotes a malignant TGFβ2 autocrine loop in glioblastoma. Cancer Discov 2014;4(10):1230–1241. https://doi.org/10.1158/2159-8290.CD-14-0275.
[44] Hua X, Zhao C, Tian J, et al. Wnt signaling dosage controlled by a Ctnnb1 enhancer balances homeostasis and tumorigenesis of intestinal epithelia. Elife 2024;13. https://doi.org/10.7554/ELIFE.98238.1.
[45] Tummala KS, Brandt M, Teijeiro A, et al. Hepatocellular carcinomas originate predominantly from hepatocytes and benign lesions from hepatic progenitor cells. Cell Rep 2017;19(3):584–600. https://doi.org/10.1016/J.CELREP.2017.03.059.
[46] Pradhan-Sundd T, Zhou L, Vats R, et al. Dual catenin loss in murine liver causes tight junctional deregulation and progressive intrahepatic cholestasis. Hepatology 2018;67(6):2320–2337. https://doi.org/10.1002/HEP.29585.

Keywords: MCPIP1; *Zc3h12a*; HCC; fibrosis; EMT; β-catenin; CREB1.
*Received 16 May 2025; received in revised form 20 January 2026; accepted 22 January 2026; Available online 29 January 2026*

Journal of Hepatology, Volume *8*

## Supplemental information

# The endonuclease MCPIP1 protects against liver cancer development in a sex-dependent manner by modulating $\beta$-catenin and CREB1

**Oliwia Kwapisz, Paulina Marona, Judyta Gorka, Rafał Myrczek, Ester Gonzalez-Sanchez, Esther Bertran, Jerzy Kotlinowski, Maciej Głuc, Ania Alay, Natalia Pydyn, Monika Kujdowicz, Emilio Ramos, Isabel Fabregat, and Katarzyna Miekus**

# The endonuclease MCPIP1 protects against liver cancer development in a sex-dependent manner by modulating β-catenin and the transcription factor CREB1

Oliwia Kwapisz, Paulina Marona, Judyta Gorka, Rafał Myrczek, Ester Gonzalez-Sanchez, Esther Bertran, Jerzy Kotlinowski, Maciej Głuc, Ania Alay, Natalia Pydyn, Monika Kujdowicz, Emilio Ramos, Isabel Fabregat, Katarzyna Miękus

Table of contents

Supplementary materials and methods....2

Table S1 ....6

Table S2 ....8

Fig. S1 ....12

Fig. S2 ....14

Fig. S3 ....16

Supplementary references ....17

Editing certificates....18

## Supplementary materials and methods

### Immunohistochemical staining (paraffin)

To visualize liver morphology, paraffin sections were stained with Picrosirius Red and a Trichrome Stain Kit (Sigma–Aldrich). For β-catenin and c-Met staining, specific antibodies (Supplementary Table S1) and EnVision Detection System Peroxidase/DAB, Rabbit/Mouse (Dako, Agilent Technologies, Santa Clara, CA, USA) were used. All images were taken using a Leica DM6 B fluorescence microscope with 5×, 10× and 20× objectives with Leica LAS X image acquisition software.

### Immunohistochemical staining (frozen)

Fresh tissues were prefixed in pure buffered formaldehyde (Chempur), washed in PBS, incubated for 12 h in 30% sucrose at 4 °C and embedded in OCT (VWR Chemicals). Then, 8-µm-thick sections were cut using a cryostat (Leica) and placed on poly-L-lysine-coated slides. Next, the sections were permeabilized (0.1% Triton X-100 in PBS) and blocked in blocking buffer (5% horse serum + 1% BSA in PBS) at room temperature for 1 h. The sections were incubated with a primary antibody in 1% BSA in PBS in a cold room. The following day, the sections were washed with PBS and incubated for 1 h at room temperature with a secondary

antibody conjugated with Alexa Fluor 488 or Alexa Fluor 546 (1:1000; Thermo Fisher Scientific) and Hoechst nuclear stain. The sections were mounted with Dako Fluorescent Mounting Medium (Agilent Technologies, cat. no. CS70330-2). Images were acquired with a Leica DM6 B fluorescence microscope with a 20× dry objective and Leica LAS X image acquisition software.

**Immunofluorescence staining of cultured hepatocytes**

Primary hepatocytes were seeded onto 1% collagenase-coated coverslips with a thickness of 0.17 mm, placed in the wells of a 6-well plate and fixed in 4% paraformaldehyde (Chempur) for 48 h. Coverslip cultures were permeabilized with 1% Triton X-100 in phosphate-buffered saline (PBS) and blocked with 0.2% Triton X-100 (Sigma–Aldrich) in 1% BSA in PBS. The cells were incubated with primary antibodies against β-catenin S552 (1:250), β-catenin S675 (1:100) and Creb S133 (1:250) in PBS with 1% BSA at 4°C overnight and with secondary antibodies against AlexaFluor 488 and 647 (1:1000; Thermo Fisher Scientific) for 1 h in the dark at room temperature. DNA counterstaining was performed with DAPI (Thermo Fisher Scientific). Cell imaging was conducted using a Leica Stellaris 5 confocal microscope (Leica Microsystems, Mannheim, Germany) equipped with a 63x objective (oil immersion; numerical aperture, 1.4). Alexa Fluor 647 fluorescence was excited by a 638 nm argon laser, and emission was captured using a photomultiplier that collected light within the range of 643 to 750 nm. Alexa Fluor 488 fluorescence was excited by a 488 nm argon laser, and emission was captured using a photomultiplier that collected light within the range of 502 to 712 nm. The settings for DAPI were as follows: 405 nm for excitation and 425–502 nm for emission. The confocal pinhole diameter was set to 1 Airy unit.

**RNA isolation and qRT-PCR**

RNA was isolated from tumor tissues using fenozol (phenol–chloroform extraction, A&A Biotechnology, Gdańsk, Poland, cat. no. 203-100). The concentration of total RNA was assessed using a NanoDrop 2000 spectrophotometer (Thermo Fisher Scientific). Reverse transcription was performed using 1 mg of total RNA, oligo(dT) 15 primer (1 µg/µl, Promega, Madison, WI, USA, cat. no. C1101), dNTPs (10 mM, Promega, cat. no. U1330) and M-MLV reverse transcriptase (Promega, cat. no. M1701). Real-time PCR was carried out using SYBRGreen Master Mix (A&A Biotechnology, cat. no. 2008-1000A) and QuantStudio 3 (Applied Biosystems, Waltham, MA, USA). Gene expression was normalized to the expression of elongation factor-2. The relative levels of transcripts were quantified by the ΔCt method. The sequences of primers (Sigma-Aldrich) and annealing temperatures are listed in

Supplementary Table S2. Additionally, for RNA-seq samples, we performed clean-up and DNAse treatment according to the manufacturer's protocol (Zymo Research).

**Western blot analysis**

The liver tissue was homogenized in RIPA buffer with protease and phosphatase inhibitors. Lysates were centrifugated for 20 minutes, 11,000 × *g* at 4°C.

Cellular fractionation from $1 \times 10^6$ hepatocytes was performed by adding hypotonic buffer (50 mM HEPES, pH 7.5, 10 mM KCl, 350 mM sucrose, 1 mM EDTA, 1 mM DTT and 0.1% Triton X-100) and incubated on ice for 10 min. After 5 min of centrifugation at 2,000 × g, the supernatant was collected as the cytoplasmic fraction. Nuclear lysate was obtained after resuspended the residual pellet in lysis buffer (10 mM HEPES, pH 7.0, 100 mM KCl, 5 mM MgCl2, 0.5% NP-40, 10 µM DTT and 1 mM PMSF). Cytoplasmic and nuclear suspension were centrifuged for 20 minutes, 14000 × rpm at 4°C to receive a high-quality nuclear and cytoplasmic fraction. SDS-PAGE was conducted with a 10% polyacrylamide gel. After wet transfer to polyvinylidene difluoride membranes (Millipore, cat. no. IPVH00010), the membranes were blocked in 3% BSA in Tris-buffered saline with 0.1% Tween 20 (Sigma-Aldrich). Next, the membranes were incubated with primary antibodies at 4 °C overnight with gentle agitation. On the following day, the membranes were washed three times for 10 min with TBS with 0.1% Tween 20 and incubated with a secondary antibody for 1 h at room temperature (RT) with gentle rocking. Chemiluminescence was detected after a 5-min incubation with Immobilon Western HRP substrate (Millipore, cat no. WBKLS0050) using a ChemiDoc system (Bio-Rad). All antibodies and dilutions are listed in Supplementary Table S1.

**ELISA**

Mouse DuoSet ELISA kit for IL6 (R&D Systems, cat no. DY406) were used to evaluate the levels of secreted Il6 in mice plasma, according to the manufacturer's protocols. The absorbance was measured at 450 nm with a reference wavelength of 540 nm using a Tecan Spectre Fluor Plus microplate reader. Three independent experiments were performed in triplicate for conditioned medium experiments and without replicates for each mouse sample.

**RNA-seq analysis**

The transcriptome analysis was prepared by NOVOGENE (Cambridge, UK). RNA sequencing libraries were prepared using mRNA isolated from total RNA via poly-T oligo-attached magnetic beads. The mRNA was fragmented and converted to cDNA using random hexamer primers. Libraries were prepared using an unstranded workflow, including steps of end repair,

A-tailing, adapter ligation, size selection, amplification, and purification. Sequencing was performed on the NovaSeq X Plus platform (PE150). Original results have been deposited in the Sequence Read Archive (SRA) database.

**Gene Set Enrichment Analysis**

Gene Set Enrichment Analysis (GSEA) [1] was conducted using the GSEApy Python package [2]. The analysis was performed using a DESeq2-normalized RNA-seq dataset. The Mouse MSigDB Hallmark gene (v2023.2) set was employed for enrichment analysis [3].

**Table S1**

| Antibody | Producent | Catalog number | Dilution |
|---|---|---|---|
| β-actin | Sigma-Aldrich | 1978 | 1:2000 |
| a-tubulin | Calbiochem | CP06 | 1:1000 |
| Histone H3 | Millipore | 05-928 | 1:1000 |
| NFκB | Cell Signaling Technology | 4764 | 1:1000 |
| phospho NFκB (Ser536) | Cell Signaling Technology | 3033 | 1:1000 |
| STAT3 | Cell Signaling Technology | 4904s | 1:1000 |
| phospho STAT3 (Tyr705) | Cell Signaling Technology | 9145s | 1:1000 |
| p38 | Cell Signaling Technology | 9212S | 1:1000 |
| p38 (Thr180/Tyr182) | Cell Signaling Technology | 9211S | 1:1000 |
| Akt | Cell Signaling Technology | 9272 | 1:1000 |
| Akt (Ser473) | Cell Signaling Technology | 4060S | 1:1000 |
| ERK1/2 (T202/Y204) | Cell Signaling Technology | 4370 | 1:1000 |
| MAPK (ERK1/2) | Cell Signaling Technology | 9102 | 1:1000 |
| Non-phospho β-catenin (Ser45) | Cell Signaling Technology | 19807 | 1:1000 |
| β-catenin (Ser675) | Cell Signaling Technology | 4176S | 1:1000 |
| β-catenin (Ser552) | Cell Signaling Technology | 9566 | 1:1000 |

| | | | |
|---|---|---|---|
| CREB1 (Ser133) | Cell Signaling Technology | 9198S | 1:1000 |
| Met (Y1234/1235) | Cell Signaling Technology | 3077 | 1:1000 |
| Met | Santa Cruz | Sc-10 | 1:1000 |
| CD45 | Cell Signaling Technology | 70257 | 1:100 |
| a-SMA | Cell Signaling Technology | 48938S | 1:100 |
| CD68 | Abcam | Ab125212 | 1:100 |
| Yes1 | Cell Signaling Technology | 3201S | 1:1000 |
| RhoA | Cell Signaling Technology | 2117S | 1:1000 |
| Yap | Cell Signaling Technology | 14074 | 1:1000 |
| Glutamine Synthetase | Proteintech | 66323-2-Ig | 1:100 |
| Anty-rabbit IgG-HRP | Santa Cruz Biotechnology | sc-2357 | 1:4000 |
| Anty-mouse IgG-HRP | Santa Cruz Biotechnology | sc-516102 | 1:4000 |

**Table S2**

| | |
|---|---|
| *ZC3H12A* | For 5’- GGAAGCAGCCGTGTCCCTATG |
| | Rev 5’ - TCCAGGCTGCACTGCTCACTC |
| *CTNNB1* | For 5’- AAAATGGCAGTGCGTTTAG |
| | Rev 5’ - TTTGAAGGCAGTCTGTCGTA |
| *TGFB2* | For 5’- AGATTTGCAGGTATTGATGG |
| | Rev 5’ - ATTTCTAAAGCAATAGGCCG |
| *SPP1* | For 5’- GACCAAGGAAAACTCACTAC |
| | Rev 5’ - CTGTTTAACTGGTATGGCAC |
| *Zc3h12a* | For 5’- CAGCCTCGACCAGATGTGCC |
| | Rev 5’ - CAGCCGCTCCTCGATGAAGC |
| *Ef2* | For 5’ - GACATCACCAAGGGTGTGCAG |
| | Rev 5’ - TTCAGCACACTGGCATAGAGGC |
| *Vim* | For 5’- GAACCTGAGAGAAACTAACC |
| | Rev 5’ - GATGCTGAGAAGTCTCATTG |
| *Ctnnb1* | For 5’- GATTAACTATCAGGATGACGC |
| | Rev 5’- TTATTAACTACCACCTGGTCC |
| *Hgf* | For 5’ - CAAATGCAAGGACCTTAGAG |
| | Rev 5’- CTTGTTTTGGATAAGTTGCC |
| *Fn1* | For 5’ - CCTATAGGATTGGAGACACG |
| | Rev 5’- GTTGGTAAATAGCTGTTCGG |

| | |
|---|---|
| *Il1b* | For 5’ - GGATGATGATGATAACCTGC |
| | Rev 5’- CATGGAGAATATCACTTGTTGG |
| *Cxcl12* | For 5’ - GAAAGCTTTAAACAAGAGGC |
| | Rev 5’- GTGAAAGTACAGCAAAACTG |
| *Cxcr4* | For 5’ - ATACTCACACTGATCGGTTC |
| | Rev 5’- AGGTAGCAGTGAAACCTC |
| *Mmp2* | For 5’ - GAGATCTTCTTCTTCAAGGAC |
| | Rev 5’- AATAGACCCAGTACTCATTCC |
| *Zeb1* | For 5’ - ATATGAGCACACAGGTAAGAG |
| | Rev 5’- TTCATGTGTTGAGAGTAGGAG |
| *Twist* | For 5’ - GAGACCTAGATGTCATTGTTTC |
| | Rev 5’- GAATTTGGTCTCTGCTCTTC |
| *Ctgf* | For 5’ - GAGGAAAACATTAAGAAGGGC |
| | Rev 5’- AGAAAGCTCAAACTTGACAG |
| *Spp1* | For 5’ - GGATGAATCTGACGAATCTC |
| | Rev 5’- GCATCAGGATACTGTTCATC |
| *Adam17* | For 5’ - AGCTTATTACAACCCAACTG |
| | Rev 5’- CAGCTTCCTTTGTGAGAATAG |
| *Creb1* | For 5’ - TGTGTTACGTGGGGGAGAGAA |
| | Rev 5’- CATGGATACCTGGGCTAATGTGG |
| *Tgfb2* | For 5’ - GAGATTTGCAGGTATTGATGG |

| | |
|---|---|
| | Rev 5'- CAACAACATTAGCAGGAGATG |
| *Src* | For 5' - AATAACACAGAGGGAGACTG |
| | Rev 5' - ATTCCCGTCTAGTGATCTTG |
| *Myc* | For 5' - TTTTGTCTATTTGGGGACAG |
| | Rev 5' - CATAGTTCCTGTTGGTGAAG |
| *Il6* | For 5' - ACTTCACAAGTCGGAGGCTT |
| | Rev 5' - GGTACTCCAGAAGACCAGAGG |
| *Cd3e* | For 5' - ATCTTGGTAGAGAGAGCATTC |
| | Rev 5' - CCCATTTTAAGTTCTCGTCAC |
| *Cd14* | For 5' - CTCTGTCCTTAAAGCGGCTTAC |
| | Rev 5' - GTTGCGGAGGTTCAAGATGTT |
| *Mgl2* | For 5' - AGGCACCCTAAGAGCCATTT |
| | Rev 5' - CCCTCTTCTCCAGTGTGCTC |
| *Wnt4* | For 5' - GTCAGGATGCTCGGACAACAT |
| | Rev 5' - CACGTCTTTACCTCGCAGGA |
| *Wnt5* | For 5' - CAACTGGCAGGACTTTCTCAA |
| | Rev 5' - CATCTCCGATGCCGGAACT |
| *Wnt6* | For 5' - GCAAGACTGGGGGTTCGAG |
| | Rev 5' - CCTGACAACCACACTGTAGGAG |
| *Wnt7* | For 5' - TGAACTTACACAATAACGAGGCG |
| | Rev 5' - GTGGTCCAGCACGTCTTAGT |

| | |
|---|---|
| *Wnt11* | For 5’ - ATGCGTCTACACAACAGTGAAG |
| | Rev 5’ - GTAGCGGGTCTTGAGGTCAG |
| *Itpr3* | For 5’ - AAGTACGGCAGCGTGATTCAG |
| | Rev 5’ - CACGACCACATTATCCCCATTG |
| *Tbx3* | For 5’ - GAACCTACCTGTTCCCGGAAA |
| | Rev 5’ - AGTGTCTCGAAAACCCTTTGC |
| *Fzd8* | For 5’ - ATGGAGTGGGTTACCTGTTG |
| | Rev 5’ - CACCGTGATCTCTTGGCAC |
| *Dvl2* | For 5’ - ATGGATCAGGATTTTGGGGTGG |
| | Rev 5’ - GTGGGGTATCTGACGACACAA |
| *Axin* | For 5’ - ATGAGTAGCGCCGTGTTAGTG |
| | Rev 5’ - GGGCATAGGTTTGGTGGACT |
| *Tnfa* | For 5’ - AATTCGAGTGACAAGCCTGTAGCC |
| | Rev 5’ - TGTCTTTGAGATCCATGCCGTTGG |
| *Ifng* | For 5’ - CAGCAAGGCGAAAAAGGATG |
| | Rev 5’ - CAGATACAACCCCGCAATCA |
| *Csf2* | For 5’ - TGACAGCCAGCTACTACCAG |
| | Rev 5’ - TCATTACGCAGGCACAAAAGC |
| *Casp1* | For 5’ - CCCACTGCTGATAGGGTGAC |
| | Rev 5’ - GCATAGGTACATAAGAATGAACTGGA |

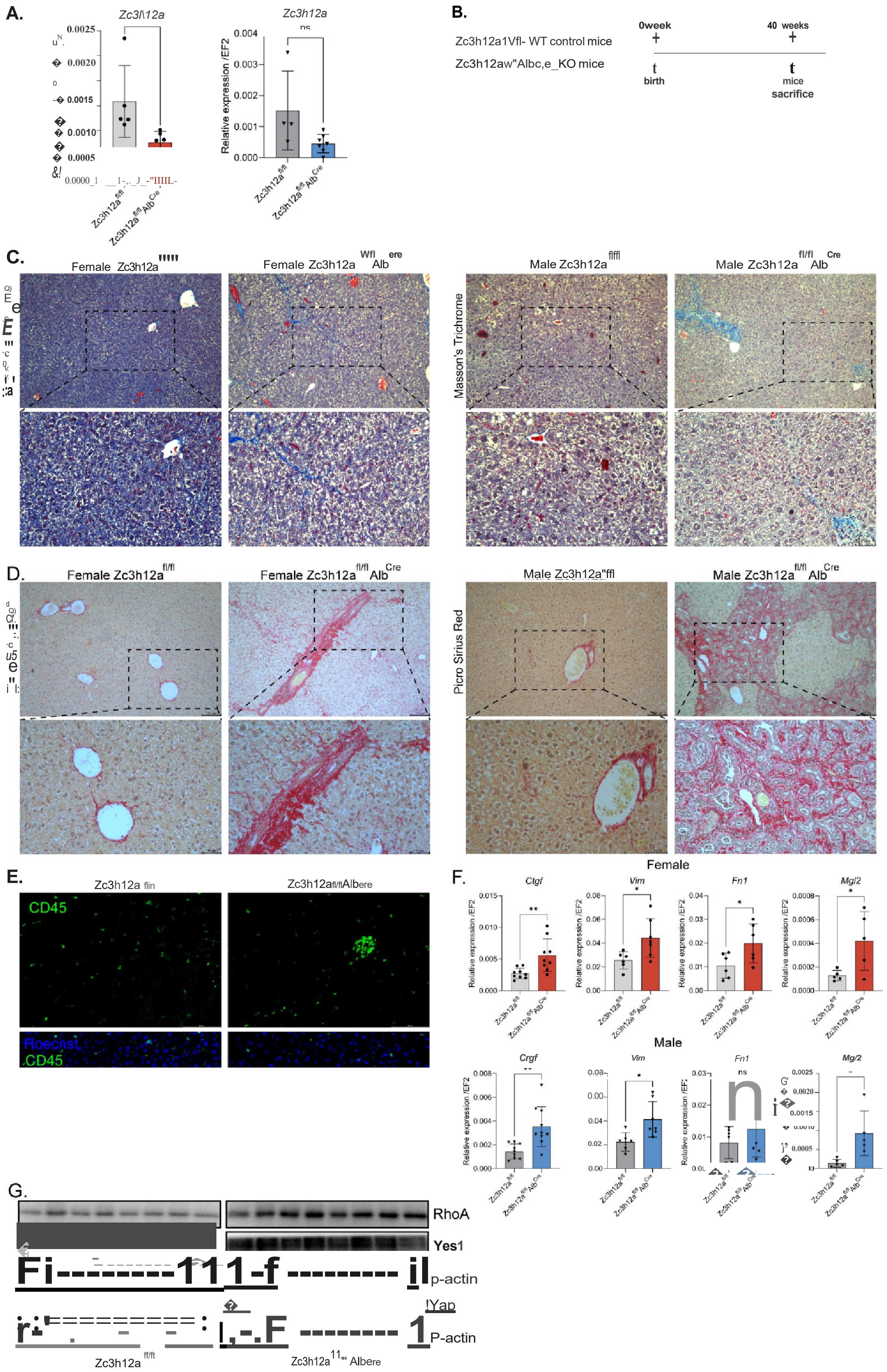
A.
Zc3h12a
Relative expression /EF2
0.0025
0.0020
0.0015
0.0010
0.0005
0.0000
0.004
0.003
0.002
0.001
0.000
ns
Zc3h12a fl/fl
Zc3h12a fl/fl Alb Cre
B.
Zc3h12a fl/fl WT control mice
Zc3h12a fl/fl Alb Cre KO mice
0 week
40 weeks
birth
mice sacrifice
C.
Female Zc3h12a fl/fl
Female Zc3h12a fl/fl Alb Cre
Male Zc3h12a fl/fl
Male Zc3h12a fl/fl Alb Cre
Masson's Trichrome
D.
Female Zc3h12a fl/fl
Female Zc3h12a fl/fl Alb Cre
Male Zc3h12a fl/fl
Male Zc3h12a fl/fl Alb Cre
Picro Sirius Red
E.
Zc3h12a fl/fl
Zc3h12a fl/fl Alb Cre
CD45
Hoechst
CD45
F.
Female
Ctgf
Vim
Fn1
Mgl2
Male
Ctgf
Vim
Fn1
Mgl2
Relative expression /EF2
ns
G.
RhoA
Yes1
β-actin
Yap
β-actin
Zc3h12a fl/fl
Zc3h12a fl/fl Alb Cre

**Fig. S1. *Zc3h12a* knock out in the liver leads to increased levels of factors involved in EMT and fibrosis. A -** mRNA expression level of *Zc3h12a* in 42-weeks old male and female mice. *EF2* was used as the reference gene, N = 4-7 per group. The results are presented as the mean ± SD with dot plot. *P* values were estimated using unpaired Student's *t*-test or U Mann-Whitney $**P < 0.01$.**B -** Schematic representation of the *in vivo* model without DEN administration. **C, D -** Representative images of Masson's trichrome and Picro Sirius Red staining for liver $Zc3h12a^{fl/fl}Alb^{Cre}$ and $Zc3h12a^{fl/fl}$ 42 weeks old male and female mice. **E** – Representative images of CD45 immunofluorescent staining of liver 42 weeks old $Zc3h12a^{fl/fl}Alb^{Cre}$ mice and $Zc3h12a^{fl/fl}$ control mice (Hoechst for nuclei; CD45 antibody labeled with fluorescent dye AlexaFluor 488). **F -** mRNA expression level of *Ctgf, Vim, Fn1,* and *Mgl2*. *EF2* was used as the reference gene. 42-weeks old males and females N = 5-10 per group. The results are presented as the mean ± SD with dot plot. *P* values were estimated using unpaired Student's *t*-test or U Mann-Whitney $*P < 0.05$, $**P < 0.01$. **G** – Western blot analysis of Yes1, RhoA and Yap in 42-weeks old mice with β-actin as the loading control.

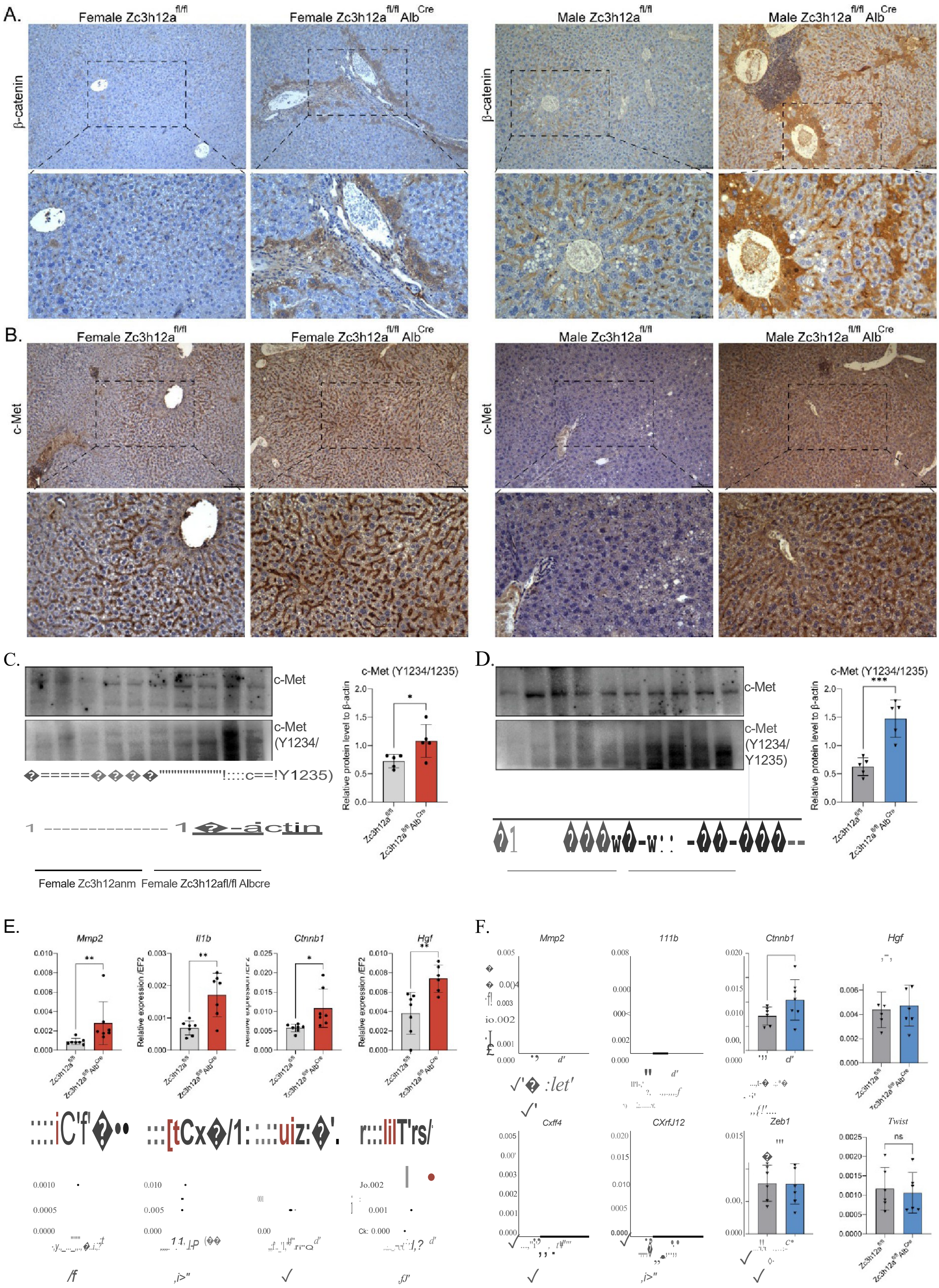
A.
Female Zc3h12a fl/fl
Female Zc3h12a fl/fl Alb Cre
Male Zc3h12a fl/fl
Male Zc3h12a fl/fl Alb Cre
β-catenin
B.
c-Met
C.
c-Met
c-Met (Y1234/
Y1235)
β-actin
c-Met (Y1234/1235)
Relative protein level to β-actin
Female Zc3h12a fl/fl
Female Zc3h12afl/fl Albcre
D.
c-Met
c-Met (Y1234/ Y1235)
E.
Mmp2
Il1b
Ctnnb1
Hgf
Relative expression /EF2
F.
Mmp2
Ctnnb1
Hgf
Cxcl4
Zeb1
Twist
ns

**Fig. S2. β-catenin and c-Met level is affected by Zc3h12a.**

**A -** Representative images of β-catenin IHC staining of liver Zc3h12a$^{fl/fl}$Alb$^{Cre}$ and Zc3h12a$^{fl/fl}$ mice (female and male). **B** – Representative images of C-Met IHC staining of liver Zc3h12a$^{fl/fl}$Alb$^{Cre}$ and Zc3h12a$^{fl/fl}$ mice (female and male). **C, D** – Analysis of c-Met and phospho-c-Met (Y1234/Y1235) protein level in female (C, N = 5 per group) and male (D, N = 5 per group) Zc3h12a$^{fl/fl}$Alb$^{Cre}$ and Zc3h12a$^{fl/fl}$ cmice. Graphs represent densitometric quantification with β-actin as the loading control. P values were estimated using two-tailed unpaired Student's *t*-test, *P < 0.05, ***P < 0.001. **E, F** – mRNA expression level of *Mmp2, Ctnnb1, Hgf, Zeb1, Twist*, *Il1b, Cxcl12,* and *Cxcr4*. *EF2* was used as the reference gene. Females N = 5-9 per group, males N = 5-10 per group. The results are presented as the mean ± SD with dot plot. *P* values were estimated using unpaired Student's *t*-test or U Mann-Whitney **P* < 0.05, ***P* < 0.01.

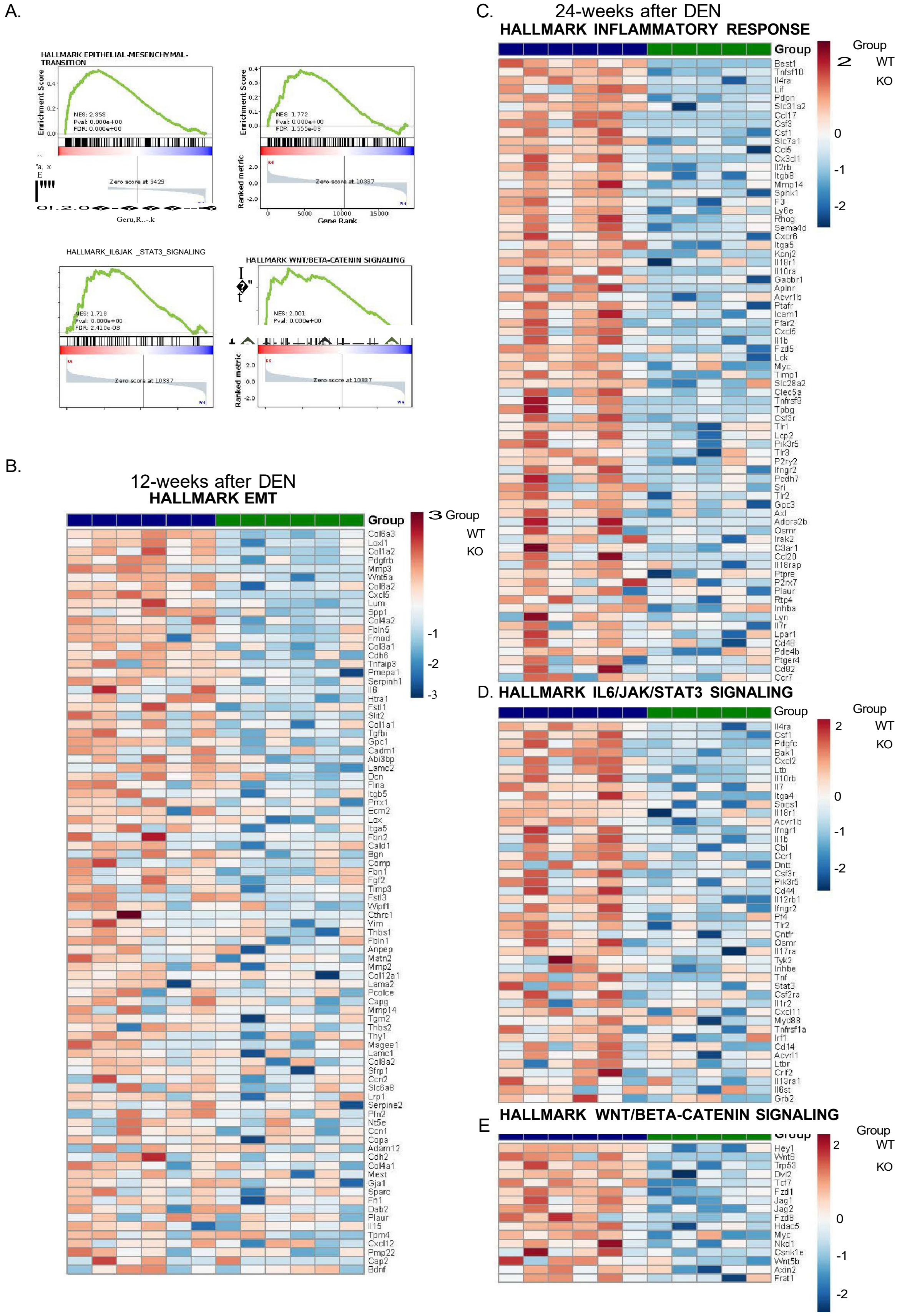
A.
HALLMARK EPITHELIAL-MESENCHYMAL-TRANSITION
Enrichment Score
NES: 2.353
Pval: 0.000e+00
FDR: 0.000e+00
Zero score at 9429
Enrichment Score
NES: 1.772
Pval: 0.000e+00
FDR: 1.555e-03
Ranked metric
Zero score at 10337
Gene Rank
HALLMARK_IL6JAK _STAT3_SIGNALING
NES: 1.718
Pval: 0.000e+00
FDR: 2.410e-03
Zero score at 10337
HALLMARK WNT/BETA-CATENIN SIGNALING
NES: 2.001
Pval: 0.000e+00
Ranked metric
Zero score at 10337
B.
12-weeks after DEN
HALLMARK EMT
Group
WT
KO
Col6a3 Loxl1 Col1a2 Pdgfrb Mmp3 Wnt5a Col6a2 Cxcl5 Lum Spp1 Col4a2 Fbln5 Fmod Col3a1 Cdh6 Tnfaip3 Pmepa1 Serpinh1 Il6 Htra1 Fstl1 Slit2 Col1a1 Tgfbi Gpc1 Cadm1 Abi3bp Lamc2 Dcn Flna Itgb5 Prrx1 Ecm2 Lox Itga5 Fbn2 Cald1 Bgn Comp Fbn1 Fgf2 Timp3 Fstl3 Wipf1 Cthrc1 Vim Thbs1 Fbln1 Anpep Matn2 Mmp2 Col12a1 Lama2 Pcolce Capg Mmp14 Tgm2 Thbs2 Thy1 Magee1 Lamc1 Col8a2 Sfrp1 Ccn2 Slc6a8 Lrp1 Serpine2 Pfn2 Nt5e Ccn1 Copa Adam12 Cdh2 Col4a1 Mest Gja1 Sparc Fn1 Dab2 Plaur Il15 Tpm4 Cxcl12 Pmp22 Cap2 Bdnf
-1
-2
-3
C.
24-weeks after DEN
HALLMARK INFLAMMATORY RESPONSE
Group
WT
KO
0
-1
-2
Best1 Tnfsf10 Il4ra Lif Pdpn Slc31a2 Ccl17 Csf3 Csf1 Slc7a1 Ccl5 Cx3cl1 Il2rb Itgb8 Mmp14 Sphk1 F3 Ly6e Rhog Sema4d Cxcr6 Itga5 Kcnj2 Il18r1 Il10ra Gabbr1 Aplnr Acvr1b Ptafr Icam1 Ffar2 Cxcl5 Il1b Fzd5 Lck Myc Timp1 Slc28a2 Clec5a Tnfrsf9 Tpbg Csf3r Tlr1 Lcp2 Pik3r5 Tlr3 P2ry2 Ifngr2 Pcdh7 Sri Tlr2 Gpc3 Axl Adora2b Osmr Irak2 C3ar1 Ccl20 Il18rap Ptpre P2rx7 Plaur Rtp4 Inhba Lyn Il7r Lpar1 Cd48 Pde4b Ptger4 Cd82 Ccr7
D. HALLMARK IL6/JAK/STAT3 SIGNALING
Group
WT
KO
2
0
-1
-2
Il4ra Csf1 Pdgfc Bak1 Cxcl2 Ltb Il10rb Il7 Itga4 Socs1 Il18r1 Acvr1b Ifngr1 Il1b Cbl Ccr1 Dntt Csf3r Pik3r5 Cd44 Il12rb1 Ifngr2 Pf4 Tlr2 Cntfr Osmr Il17ra Tyk2 Inhbe Tnf Stat3 Csf2ra Il1r2 Cxcl11 Myd88 Tnfrsf1a Irf1 Cd14 Acvrl1 Ltbr Crlf2 Il13ra1 Il6st Grb2
E
HALLMARK WNT/BETA-CATENIN SIGNALING
Group
WT
KO
2
0
-1
-2
Hey1 Wnt6 Trp53 Dvl2 Tcf7 Fzd1 Jag1 Jag2 Fzd8 Hdac5 Myc Nkd1 Csnk1e Wnt5b Axin2 Frat1

**Fig. S3. GSEA Plots and leading edge genes for Selected gene sets**

**A** - GSEA plots for selected gene sets from an analysis performed against the mouse Hallmark gene sets from MSigDB. The plots display enrichment for upregulated pathways in knockout mice. The y-axis represents the enrichment score (ES), while the x-axis indicates the ranked position of all genes included in the analysis. The peak of each curve corresponds to the maximum ES, calculated by walking down the ranked gene list. Genes contributing most to the enrichment signal before this peak are defined as the 'leading edge' subset. **B, C, D, E** - Corresponding heatmaps show the expression of leading-edge genes, based on normalized RNA-seq read counts.

## Supplementary references


1. Subramanian A, Tamayo P, Mootha VK, et al. Gene set enrichment analysis: A knowledge-based approach for interpreting genome-wide expression profiles. *Proceedings of the National Academy of Sciences*. 2005;102(43):15545-15550. doi:10.1073/pnas.0506580102
2. Fang Z, Liu X, Peltz G. GSEApy: a comprehensive package for performing gene set enrichment analysis in Python. *Bioinformatics*. 2023;39(1). doi:10.1093/bioinformatics/btac757
3. Liberzon A, Birger C, Thorvaldsdóttir H, Ghandi M, Mesirov JP, Tamayo P. The Molecular Signatures Database Hallmark Gene Set Collection. *Cell Syst*. 2015;1(6):417-425. doi:10.1016/j.cels.2015.12.004

**SPRINGER NATURE**
# Author Services

## Editing Certificate

This document certifies that the manuscript

Endonuclease MCPIP1 protects against liver cancer development in a gender-dependent manner by modulating β-Catenin and the transcription factor CREB1

prepared by the authors

Oliwia Kwapisz, Paulina Marona, Judyta Gorka, Rafał Myrczek, Ester Gonzalez-Sanchez, Esther Bertran, Jerzy Kotlinowski, Maciej Głuc, Ania Alay, Natalia Pydyn, Monika Kujdowicz, Emilio Ramos, Isabel Fabregat, Katarzyna Miekus

was edited for proper English language, grammar, punctuation, spelling, and overall style by one or more of the highly qualified English speaking editors at SNAS.

This certificate was issued on **April 29, 2025** and may be verified on the SNAS website using the verification code **3401-F1BA-84B9-4DA0-10DP** .

Neither the research content nor the authors' intentions were altered in any way during the editing process. Documents receiving this certification should be English-ready for publication; however, the author has the ability to accept or reject our suggestions and changes. To verify the final SNAS edited version, please visit our verification page at secure.authorservices.springernature.com/certificate/verify.
If you have any questions or concerns about this edited document, please contact SNAS at support@as.springernature.com.

SNAS provides a range of editing, translation, and manuscript services for researchers and publishers around the world.
For more information about our company, services, and partner discounts, please visit authorservices.springernature.com.

**SPRINGER NATURE**
# Author Services

## Editing Certificate

This document certifies that the manuscript

The endonuclease MCPIP1 protects against liver cancer development in a sex-dependent manner by modulating β-catenin and the transcription factor CREB1

prepared by the authors

Oliwia Kwapisz, Paulina Marona, Judyta Gorka, Rafał Myrczek, Ester Gonzalez-Sanchez, Esther Bertran, Jerzy Kotlinowski, Maciej Głuc, Ania Alay, Natalia Pydyn, Monika Kujdowicz, Emilio Ramos, Isabel Fabregat, Katarzyna Miekus

was edited for proper English language, grammar, punctuation, spelling, and overall style by one or more of the highly qualified English speaking editors at SNAS.

This certificate was issued on **December 17, 2025** and may be verified on the SNAS website using the verification code **CFC3-F3B2-B2C2-B9B3-C025** .

Neither the research content nor the authors' intentions were altered in any way during the editing process. Documents receiving this certification should be English-ready for publication; however, the author has the ability to accept or reject our suggestions and changes. To verify the final SNAS edited version, please visit our verification page at secure.authorservices.springernature.com/certificate/verify.
If you have any questions or concerns about this edited document, please contact SNAS at support@as.springernature.com.

SNAS provides a range of editing, translation, and manuscript services for researchers and publishers around the world.
For more information about our company, services, and partner discounts, please visit authorservices.springernature.com.
